# Phylogeny-guided microbiome OTU-specific association test (POST)

**DOI:** 10.1186/s40168-022-01266-3

**Published:** 2022-06-07

**Authors:** Caizhi Huang, Benjamin J. Callahan, Michael C. Wu, Shannon T. Holloway, Hayden Brochu, Wenbin Lu, Xinxia Peng, Jung-Ying Tzeng

**Affiliations:** 1 Bioinformatics Research Center, North Carolina State University, Raleigh, 27606 USA; 2 Department of Population Health and Pathobiology, North Carolina State University, Raleigh, 27607 USA; 3 Division of Public Health Sciences, Fred Hutchinson Cancer Research Center, Seattle, 98109 USA; 4 Department of Statistics, North Carolina State University, Raleigh, 27606 USA; 5 Department of Molecular Biomedical Sciences, North Carolina State University, Raleigh, 27607 USA

**Keywords:** Association test, Phylogenetic tree, Kernel machine regression

## Abstract

**Background:**

The relationship between host conditions and microbiome profiles, typically characterized by operational taxonomic units (OTUs), contains important information about the microbial role in human health. Traditional association testing frameworks are challenged by the high dimensionality and sparsity of typical microbiome profiles. Phylogenetic information is often incorporated to address these challenges with the assumption that evolutionarily similar taxa tend to behave similarly. However, this assumption may not always be valid due to the complex effects of microbes, and phylogenetic information should be incorporated in a *data-supervised* fashion.

**Results:**

In this work, we propose a local collapsing test called phylogeny-guided microbiome OTU-specific association test (POST). In POST, whether or not to borrow information and how much information to borrow from the neighboring OTUs in the phylogenetic tree are supervised by phylogenetic distance and the outcome-OTU association. POST is constructed under the kernel machine framework to accommodate complex OTU effects and extends kernel machine microbiome tests from community level to OTU level. Using simulation studies, we show that when the phylogenetic tree is informative, POST has better performance than existing OTU-level association tests. When the phylogenetic tree is not informative, POST achieves similar performance as existing methods. Finally, in real data applications on bacterial vaginosis and on preterm birth, we find that POST can identify similar or more outcome-associated OTUs that are of biological relevance compared to existing methods.

**Conclusions:**

Using POST, we show that adaptively leveraging the phylogenetic information can enhance the selection performance of associated microbiome features by improving the overall true-positive and false-positive detection. We developed a user friendly R package *POSTm* which is freely available on CRAN (https://CRAN.R-project.org/package=POSTm).

Video Abstract.

**Supplementary Information:**

The online version contains supplementary material available at (10.1186/s40168-022-01266-3).

## Background

The human microbiome is the collection of microorganisms residing in the human body and has been definitively shown to impact human disease and health [1]. Multiple resources are now available that enable the characterization of microbial communities in the human body and further our understanding of how the human microbiota affect clinical outcomes of the host, e.g., the Human Microbiome Project [2] and Integrative Human Microbiome Project [3]. One common strategy to determine microbial compositions is marker gene sequencing, which amplifies and sequences a fingerprint gene (e.g., 16S rRNA gene) that carries species-specific identifiers. The generated sequencing reads can be either clustered into operational taxonomic units (OTUs) based on sequence similarity [4] or denoised into amplicon sequence variants (ASVs) with exact sequences [5]. For simplicity, hereafter, we use “OTU” as a general reference to a taxonomic unit when discussing the overall concepts. We will explicitly distinguish OTU and ASV when the two terms need to be discerned, such as in the real data analysis.

Comparison of OTU profiles with respect to differential conditions or clinical outcomes lends important knowledge towards understanding the microbial roles in affecting health [1, 6], and distance-based approaches are widely adopted to evaluate such association at the community level [7–11]. It is also of interest to identify specific OTUs or organisms as biomarkers that drive the global association, which can provide crucial insights into the microbial functions and mechanisms in diseases. For example, the correlation between *Lactobacillus crispatus* abundance and host vaginal cytokine IP-10/CXCL10 levels may help to explain the induction of preterm birth [12].

OTU-level analysis can be challenging due to sparse OTU counts, low OTU abundance, and high OTU dimensionality. Multiple studies have shown that incorporating phylogenetic structure among OTUs can increase the detecting power or predictive accuracy of a microbiome analysis because phylogenetically related species or OTUs are expected to impact or respond to environmental disturbances in a similar way [13]. For example, Xiao et al. [14] introduced a false discovery rate (FDR) control procedure, TreeFDR, that uses the phylogenetic tree to specify the prior correlation among the *p*-values of individual OTUs. TreeFDR is more powerful than the classic FDR controlling methods (e.g., the Benjamini-Hochberg (BH) FDR method) as well as those that incorporate grouping structure [15, 16]. Xiao et al. [17] introduced a predictive method, glmmTree, based on a generalized linear mixed model that encourages the nearby OTUs in the phylogenetic tree to share similar effects and showed that glmmTree improves prediction performance, especially when associated OTUs are clustered on the tree. Kim et al. [18] introduced a genus-level collapsing association test, TMAT, that first assesses the association for each OTU within a genus and then uses the minimum OTU *p*-value to determine the genus significance.

However, it has been noted that inappropriate incorporation of phylogenetic information can degrade testing performance when phylogeny is not informative of association patterns among OTUs [19]. Furthermore, evolutionarily close OTUs can act differently and even in opposite ways. For example, two close species in *Lactobacillus* genus, i.e., *Lactobacillus iners* and *Lactobacillus crispatus*, exhibit opposite associations with preterm birth—*Lactobacillus crispatus* dominance was negatively associated with preterm birth while *Lactobacillus iners* dominance was positively associated with preterm birth [20].

To address these challenges in the incorporation of phylogenetic information, we propose the phylogeny-guided microbiome OTU-specific association test (POST), which boosts the detecting power of the target OTU by adaptively borrowing information from its phylogenetically close OTUs. Whether or not to borrow information and how much information to borrow from the neighboring OTUs are supervised by phylogenetic distance and the outcome-OTU association. POST is built on a kernel machine regression framework, which can flexibly accommodate complex microbiome effects, easily adjust for covariates, and be applicable to continuous and binary outcomes. Compared to existing phylogeny-informed single-OTU methods, POST can better handle sparse OTU data and allow information collapsing from OTUs of opposite effects because kernel methods collapse OTU information at similarity/kernel level instead of at the level of *p*-values or abundance counts. POST extends the current community-level kernel tests [10, 11, 21] to OTU-level test. Finally, POST is computationally efficient for OTU-wide analysis as its *p*-value can be analytically obtained. Through extensive numerical studies, we show that POST has favorable performance in identifying associated OTUs over existing methods in simulations and real data applications, including detecting the association of vaginal microbiome with bacterial vaginosis and with preterm birth.

## Methods

Suppose that for subject *i*, *i*=1,⋯,*n*, we observe the outcome value *y*_*i*_, which can be continuous or binary; the vector of *p* covariates ***x***_*i*_=(*x*_*i*1_,⋯,*x*_*ip*_)^⊤^, and the abundance vector of *M* OTUs ***z***_*i*_=(*z*_*i*1_,⋯,*z*_*iM*_)^⊤^, after sequence processing. In matrix presentation, we have the *n*×*M* matrix of OTU abundance ***Z***=(***z***_1_,***z***_2_,…,***z***_*n*_)^⊤^, the *n*×*p* covariate matrix ***X***=(***x***_1_,***x***_2_,⋯,***x***_*n*_)^⊤^, and the outcome vector ***y***=(*y*_1_,*y*_2_,⋯,*y*_*n*_)^⊤^.

POST uses a kernel machine regression [10, 22] to model the relationship between OTU *m* and the outcome variable:

1 $$ g(\boldsymbol{\mu}) = \boldsymbol{X}\boldsymbol{\gamma}+h^{m}(\boldsymbol{Z}),   $$

where ***μ***=*E*(***y***|***X***,***Z***), the conditional expectation of ***y*** given the microbiome and covariate information; *g*(·) is the link function, which can be set to be the identity function for continuous outcomes and the logistic function for binary outcomes; ***γ*** is the *p*×1 vector of covariate regression coefficients; *h*^*m*^(·) is a smooth function in a reproducing kernel Hilbert space generated by a kernel function *k*^*m*^(***z***_*i*_,***z***_*j*_). Function *h*^*m*^(·) characterizes the effect of OTU *m* and can be specified by *k*^*m*^(***z***_*i*_,***z***_*j*_) using the dual representation of a function in kernel methods, i.e., $h^{m}(\boldsymbol {z}_{i})=\sum _{j=1}^{n}\alpha ^{m}_{j} k^{m}(\boldsymbol {z}_{i},\boldsymbol {z}_{j})$ with $\alpha ^{m}_{1},\cdots,\alpha ^{m}_{n}$ the unknown parameters. The association between OTU *m* and the outcome can be evaluated by testing *H*_0_:*h*^*m*^(***Z***)=***0***.

**Fig. 1:**
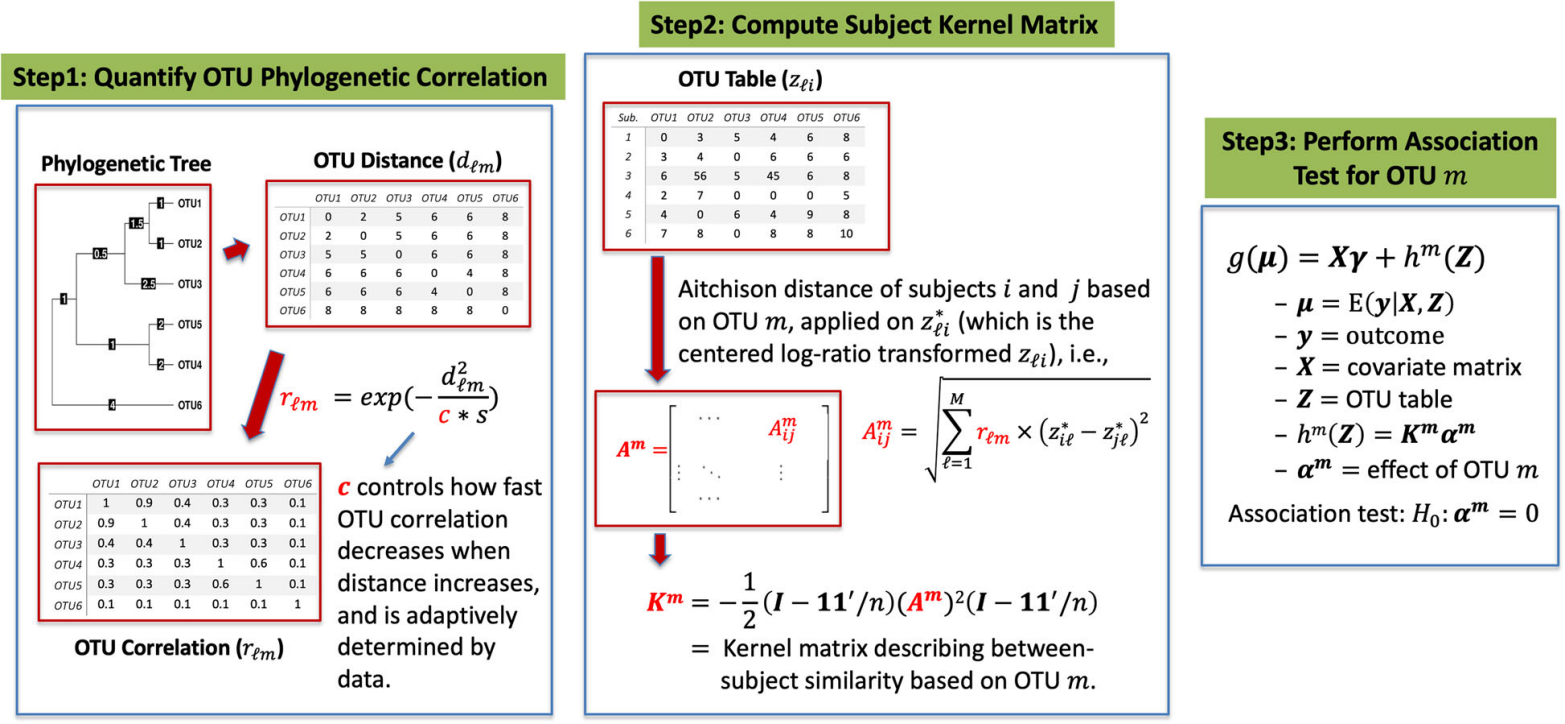
Overview of phylogeny-guided microbiome OTU-specific association test (POST)

In POST, instead of using a global kernel as in the community-level test, we propose an OTU-specific kernel function *k*^*m*^(·,·) for OTU *m* to permit the local OTU test to borrow information from its neighboring OTUs in the phylogenetic tree. Figure 1 describes the key steps of POST: (1) to quantify the pairwise OTU correlation based on their phylogenetic relationship, (2) to compute the subject kernel matrix ***K***^*m*^ for OTU *m* using OTU abundance and OTU correlation data, and (3) to evaluate the association of OTU *m* with the outcome variable using the local kernel test that seeks for the optimal amount of information borrowing. The detail of each step is described below.

### Step 1: Quantifying OTU phylogenetic correlation

**Fig. 2:**
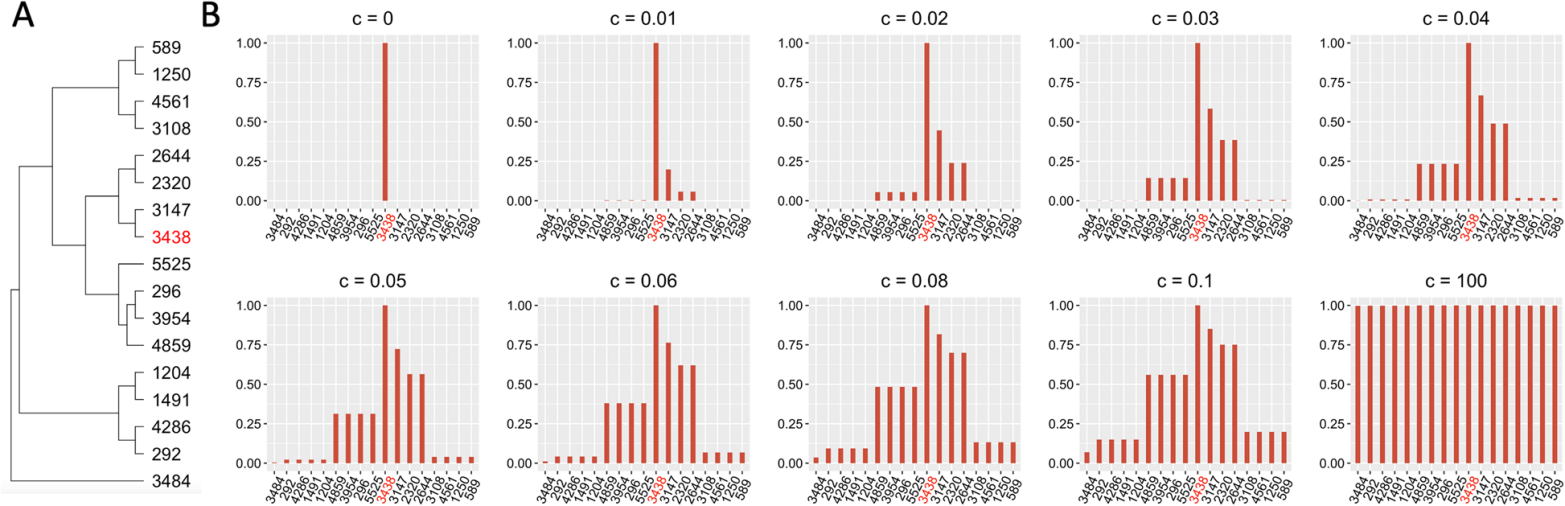
**A** Phylogenetic relationship between the target OTU (OTU3438) and its neighboring OTUs. **B** OTU phylogenetic correlation *r*_*ℓm*_ under different values of the tuning parameter *c*, illustrated by setting *m* = OTU3438 as the target OTU. The *x*-axis is the OTUs shown in the same order as the tree tips. The *y*-axis is *r*_*ℓm*_, the correlation between OTU3438 and the remaining OTUs

The first step of POST is to compute the OTU correlation matrix based on the phylogenetic relationship. To do so, we start with constructing the phylogenetic tree with *M* tips based on the sequences of the *M* OTUs using *FastTree2* [23]. We then quantify the pairwise distance between OTUs *ℓ* and *m* (denoted by *d*_*ℓm*_) by taking the sum of branch lengths from OTU *ℓ* to OTU *m* through the first common node in the tree, which can be done using the R function “cophenetic.” Finally, we convert the distance to correlation (denoted by *r*_*ℓm*_) using the Gaussian function, i.e., $r_{\ell m} = \exp \left \{-\frac {d_{\ell m}^{2}}{c\times s}\right \}$, where *s* is the standard deviation (SD) of *d*_*ℓm*_’s, *ℓ*,*m*∈{1,⋯,*M*};, and *c* is a positive data-adaptive parameter that controls how fast the OTU correlation decreases when the between-OTU distance increases. Parameter *c* is measured on the unit of distance SD so as to be scale-free and robust to trees generated from different tools (e.g., *FastTree2*, hclust). As detailed in step 2, neighboring OTUs with non-zero *r*_*ℓm*_, *ℓ*≠*m* will contribute in the association test of OTU *m*, and *r*_*ℓm*_ controls the amount of information contributed from OTU *ℓ* into the test via *c*. We illustrate in Fig. 2 a typical relationship between *c* and *r*_*ℓm*_ using the dataset of Charlson et al. [24] considered in the simulation study. Without loss of generality, we focus on OTU3438 and its 16 neighboring OTUs in the phylogenetic tree (Fig. 2A) and show the values of *r*_*ℓ*,3438_ for the neighboring OTU *ℓ* under different *c*’s in Fig. 2B. We see that smaller *c* (e.g., *c*=0.02) yields a more rapid correlation decay with increasing distance, and consequently, OTU *m* can borrow a non-negligible amount of information only from a few OTUs. In contrast, larger *c* (e.g., *c*=0.08) yields a slower correlation decay with increasing distance and defines a larger “neighborhood” of OTU *m* from which OTU *m* may borrow information. When *c*=0, *r*_*mm*_=1 and *r*_*ℓm*_=0 for all *ℓ*≠*m*, and the local association test becomes a strict single OTU test because only information of target OTU *m* is used (e.g., Fig. 2B, panel of *c*=0). When *c*=*∞*, *r*_*ℓm*_=1 for all *ℓ*, and the local association test becomes a global, community-level test because all OTUs contribute equally into the test (e.g., Fig. 2B, panel of *c*=100).

When conducting POST association test in step 3, we use data to determine the optimal *c* value. Specifically, we compute the *p*-values for a grid of *c* between 0 and *c*_max_ and use the Cauchy combination (CC) method [25, 26] to aggregate the *p*-values of different *c*’s. As detailed in the simulation studies, we set the *c*_max_=0.05 to ensure information sharing is limited within a small, concentrated neighborhood of the target OTU. The adaptively determined *c* also ensures that only informative neighbors will make contributions to the target OTU.

### Step 2: Computing subject kernel similarity

In kernel machine regression (1), one crucial component is to specify the local kernel function *k*^*m*^(***z***_*i*_,***z***_*j*_), which consequently determines the OTU effects, i.e., $h^{m}(\boldsymbol {z}_{i})=\sum _{j=1}^{n}\alpha ^{m}_{j} k^{m}(\boldsymbol {z}_{i},\boldsymbol {z}_{j})$. Several kernel choices are available for microbiome compositions in community-level tests [10, 22]. The key is to start with a distance metric to quantify the pairwise abundance dissimilarities, store them in the *n*×*n* abundance dissimilarity matrix ***A***, and then transform ***A*** to the kernel similarity matrix ***K*** using Gower’s matrix, i.e., $\boldsymbol {K}=-\frac {1}{2} \left (\boldsymbol {I}-\frac {\mathbf {1} \mathbf {1}^{\top }}{n}\right)\boldsymbol {A}^{2} \left (\boldsymbol {I}-\frac {\mathbf {1} \mathbf {1}^{\top }}{n}\right)$, where ***A***^2^ is the element-wise square of ***A***, ***I*** is the *n*×*n* identity matrix, and 1 is a vector of ones with length *n* [22]. For POST, we propose to construct a local, OTU-specific kernel ***K***^*m*^ so that only local information around the target OTU is used. First, obtain the local dissimilarity matrix ***A***^*m*^ by incorporating *r*_*ℓm*_ into the original dissimilarity matrix ***A***. Next, perform Gower’s transformation on ***A***^*m*^ to obtain $\boldsymbol {K}^{m}\equiv \left \{ k^{m}({\boldsymbol {z}_{i},\boldsymbol {z}_{j})} \right \} = -\frac {1}{2} \left (\boldsymbol {I}-\frac {\mathbf {1} \mathbf {1}^{\top }}{n}\right)(\boldsymbol {A}^{m})^{2} \left (\boldsymbol {I}-\frac {\mathbf {1} \mathbf {1}^{\top }}{n}\right)$. Although such local kernel construction can be applied on arbitrary distance metrics, in POST, we choose to use the Aitchison distance, a non-phylogenetic distance measure accounting for the compositional nature of abundance data, to avoid the potential overuse of phylogenetic information. Aitchison distance is the Euclidean distance of centered log-ratio (CLR) transformed abundance data, and the “local” Aitchison distance for OTU *m* between subjects *i* and *j* can be obtained by:

2 $$ \boldsymbol{A}_{ij}^{m} = \sqrt{\sum_{\ell=1}^{M} r_{\ell m}\times (z_{i \ell}^{*}-z_{j\ell }^{*})^{2}},   $$

where $z_{i\ell }^{*}=\log \left [\frac {z_{i\ell }}{\mathrm {G}(\boldsymbol {z}_{i})}\right ]$, i.e., the CLR transformation of the original abundance count *z*_*iℓ*_ for OTU *ℓ*, and $\mathrm {G}(\boldsymbol {z}_{i})=\sqrt [M]{z_{i1} \cdots z_{iM}}$ is the geometric mean of OTU abundance for subject *i*. A pseudo-count 0.5 is added to the OTU counts table before doing CLR transformation. If proportion data is used, a pseudo-proportion 1e −6 can be added to the proportion table [27].

### Step 3: Performing OTU-specific association test

The association of OTU *m* can be detected by testing for *H*_0_:*h*^*m*^(***Z***)=***0*** in model (1). Such a test can be constructed through the connection of kernel machine regression and generalized linear mixed models (GLMM) [28, 29]. That is, *h*^*m*^(***Z***) can be viewed as subject-specific random effects with *h*^*m*^(***Z***)∼*N*(***0***,*τ****K***^*m*^), and then testing for *H*_0_:*h*^*m*^(***Z***)=***0*** is equivalent to the variance component test of *H*_0_:*τ*=0. Under the GLMM framework, a variance component score test can be obtained as $T^{m,c} = \frac {1}{2\hat {\phi }} \left (\boldsymbol {y}-\hat {\boldsymbol {\mu }}_{0}\right)^{\top } \boldsymbol {K}^{m} \left (\boldsymbol {y}-\hat {\boldsymbol {\mu }}_{0}\right)$, where $\hat {\boldsymbol {\mu }}_{0}=g^{-1}\left (\boldsymbol {X}\hat {\boldsymbol {\gamma }}\right)$ with $\hat {\boldsymbol {\gamma }}$ the estimated covariate coefficient under the null model: *g*(***μ***)=***Xγ***, and $\hat {\phi }$ is the estimator of the dispersion parameter under *H*_0_. For continuous outcome, $\hat {\boldsymbol {\mu }}_{0}=\boldsymbol {X}\hat {\boldsymbol {\gamma }}$ and $\hat {\phi }$ equals to the estimated residual variance under *H*_0_. For binary outcome, *ϕ*=1 and $\hat {\boldsymbol {\mu }}_{0}=\exp \left \{\boldsymbol {X}\hat {\boldsymbol {\gamma }}\right \} / \left (1+\exp \left \{\boldsymbol {X}\hat {\boldsymbol {\gamma }}\right \}\right)$. With a fixed *c*, *T*^*m*,*c*^ asymptotically follows a weighted mixtures of $\chi ^{2}_{(1)}$ distribution under *H*_0_, based on which the *p*-value, denoted by *p*_*m*,*c*_, can be computed. Because the sample size tends to be moderate in microbiome studies, we use the small-sample distribution derived in Chen et al. [30] to compute *p*_*m*,*c*_. Finally, as the optimal *c* is unknown in reality, in POST, we consider a grid of *c*∈{*c*_1_,⋯,*c*_*J*_}, use the CC method [25, 26] to combine the transformed *p*-values of different *c*’s by computing $T^{m} = \sum _{j=1}^{J} \tan \left \{ \left (0.5-p_{m,c_{j}}\right)\pi \right \}$, and obtain the *p*-value of *T*^*m*^ (denoted by *p*_*m*_) by $p_{m} =\frac {1}{2}-\{\text {arctan}(T^{m}/J)\}/\pi.$ The CC method behaves like the minimum *p*-value method as *T*^*m*^ is dominated by the smallest *p*-values; yet, the CC method becomes a computationally fast alternative to the minimum *p*-value method because the *p*-value of *T*^*m*^ can be analytically obtained from a Cauchy distribution, even with correlated *p*-values [25, 26].

### Simulation study

We conducted simulation studies to evaluate the performance of POST in identifying outcome-associated OTUs. We generated OTU data based on the real dataset from the upper respiratory tract microbiome study [24], obtained from the R package *GuniFrac* [7]. The dataset consists of 856 OTU data from 60 subjects (from R object “throat.otu.tab”) and the OTU phylogenetic tree (from R object “throat.tree” constructed using *FastTree* [7]). We considered 2 different simulation settings (simulations A and B); for each simulation, we simulated OTU counts for *n*=100 individuals following Chen and Li [22] and focused on the *M*=400 most abundant OTUs in the original data. We modeled the observed OTU counts using the Dirichlet-multinomial distribution with parameters (*π*_1_,⋯,*π*_*M*_,*θ*), where *π*_*ℓ*_’s are the means of the OTU-proportions and *θ* is the over-dispersion parameter [31, 32], and obtained their maximum likelihood estimates $(\hat \pi _{1},\cdots,\hat \pi _{M}, \hat \theta)$. Next, we generated the OTU abundance proportions (*p*_1_,⋯,*p*_*M*_) from Dirichlet $(\hat \pi _{1}, \cdots,\hat \pi _{M},\hat \theta)$; we also generated the total counts of individual *i*, *N*_*i*_, from a negative binomial distribution with mean 10,000 and size 25. Finally, we generated the OTU counts of individual *i* (*z*_*i*1_,⋯,*z*_*iM*_) from multinomial (*N*_*i*_,*p*_1_,⋯,*p*_*M*_); the OTU count matrix ***Z*** comprises the resulting simulated counts.

#### Simulation A

In simulation A, we adopted the simulation design of Xiao et al. and considered a case-control study with 50 cases (i.e., *y*_*i*_=1) and 50 controls (i.e., *y*_*i*_=0). Specifically, we used the R package *pam* to partition the 400 OTUs into 20 clusters based on the phylogenetic tree and selected causal OTUs from the 8 most abundant clusters. Given ***Z*** with *M*=400 and *n*=100, we then multiplied the counts of cases with a fold change vector [ exp(*β*_1_),⋯, exp(*β*_400_)], where *β*_*m*_ is the effect size of OTU *m* generated from normal (*ν*,1) if OTU *m* is causal and 0 otherwise. We considered |*ν*| to be 1 (small effect size) or 2 (large effect size).

**Fig. 3:**
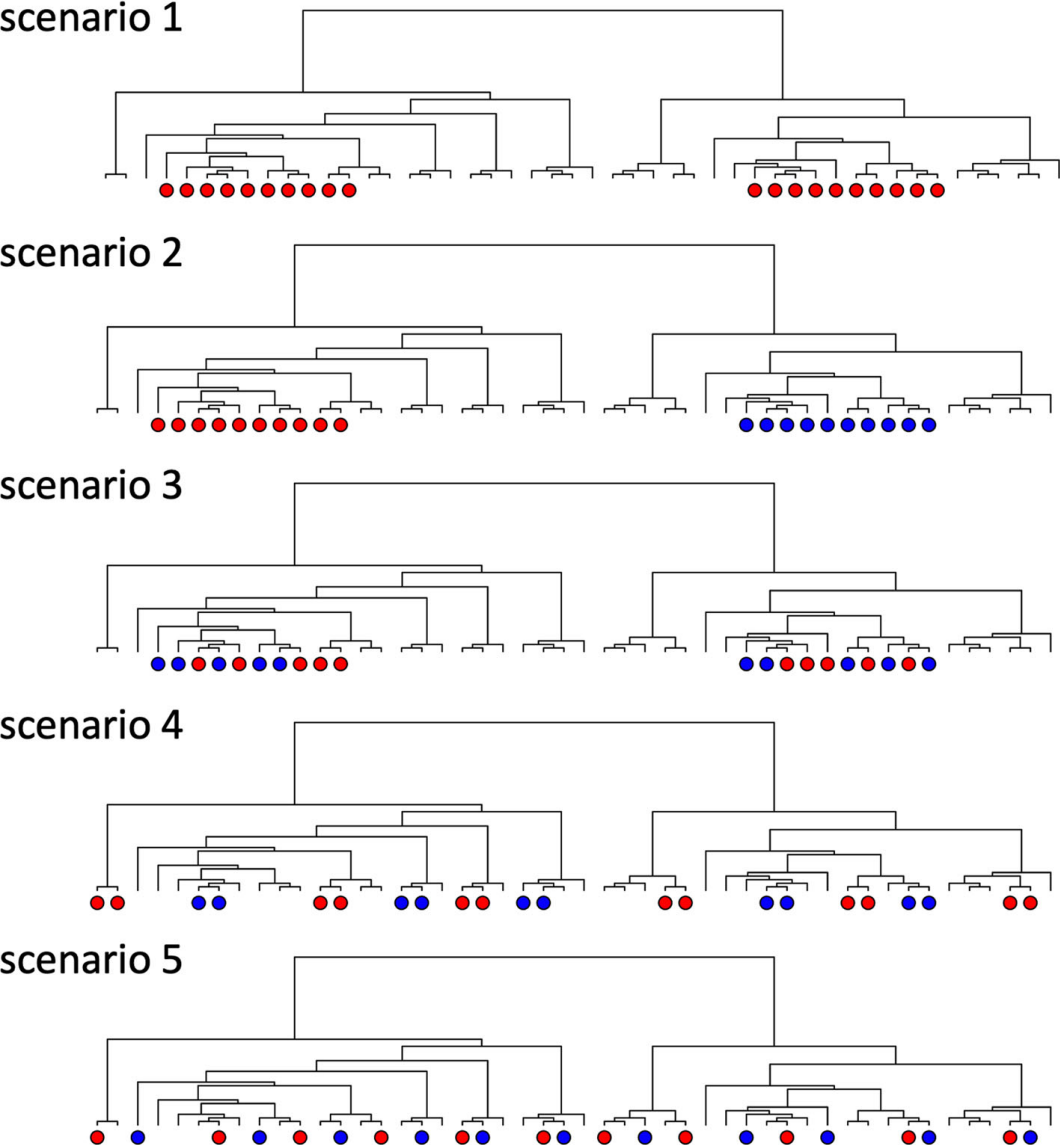
Illustration of the five causal OTU scenarios considered in the simulation. Scenarios 1 to 3 consider larger “causal hubs,” each containing about 7–10 causal OTUs; scenario 4 considers smaller causal hubs of 2–3 causal OTUs; scenario 5 considers causal OTUs with random positions in the phylogenetic tree. Red (blue) circles indicate that causal effect size tends to be positive (negative)

We consider five scenarios for causal OTUs as illustrated in Fig. 3. Scenarios 1–3 consider the same set of causal OTUs, which form multiple “causal OTU hubs” in the phylogenetic tree, with 1 causal hub from each of the top 8 abundant clusters and 7–10 causal OTUs per causal hub. In scenario 1, *β*_*m*_’s are generated from *N*(*ν*,1) for all causal OTUs. In scenario 2, *β*_*m*_∼*N*(*ν*,1) for half of the causal hubs and *β*_*m*_∼*N*(−*ν*,1) for the other half of the causal hubs. In scenario 3, a random half of the causal OTUs have their *β*_*m*_’s generated from *N*(*ν*,1), and the remaining half have their *β*_*m*_’s from *N*(−*ν*,1); consequently, a causal hub may contain a fair number of positive-effect and negative-effect causal OTUs. Scenario 4 considers the case of smaller causal hubs with 2–3 causal OTUs per hub (i.e., less-informative trees): a random half of the causal hubs with *β*_*m*_∼*N*(*ν*,1) and the other half causal hubs with *β*_*m*_∼*N*(−*ν*,1). Scenario 5 considers the case where causal OTUs are randomly chosen from the entire phylogenetic tree (i.e., non-informative trees), where a random half of the causal OTUs have *β*_*m*_∼*N*(*ν*,1) and the other half OTUs have *β*_*m*_∼*N*(−*ν*,1).

#### Simulation B

In simulation B, we considered both continuous and binary outcomes and adopted the simulation design of Zhao et al. [10] and Koh et al. [11]. Given the simulated OTU counts ***Z***={*z*_*im*_} and the same set of causal OTUs as simulation A, we generated the outcome value of individual *i* by *y*_*i*_∼*N*(*η*_*i*_,1) for continuous outcomes and *y*_*i*_∼Bernoulli(*Π*_*i*_) with $\Pi _{i}= \frac {\exp (\eta _{i}) }{1+\exp (\eta _{i})}$ for binary outcomes. Value $\eta _{i}=0.5{\omega } \times (\text {scale}(x_{1i})+\text {scale}(x_{2i})) + \sum _{m=1}^{M}\beta _{m}\times \text {scale}(z_{im})$, where *x*_1*i*_ and *x*_2*i*_ are covariates; *z*_*im*_ is the count of causal OTU *m* for subject *i*; and *ω*=1 or 0 is a parameter controlling if there are covariate effects. The “scale” function is to standardize the variable to mean 0 and standard deviation 1. Variable *x*_1*i*_ is simulated from Bernoulli(0.5) and is independent of the OTU counts, while variable *x*_2*i*_ is correlated with causal OTUs by letting *x*_2*i*_∼*N*(*δ*_*i*_,1) with $\delta _{i} =\text {scale}(\sum _{m \in \text {causal OTUs}}z_{im})$. For non-causal OTUs, *β*_*m*_=0; for causal OTUs, *β*_*m*_∼normal(*ν*,*ν*/5) or normal (−*ν*,*ν*/5) dependent on the scenario. We considered |*ν*| to be 0.2 (small effect size) and 0.5 (large effect size) for continuous outcomes and to be 0.3 (small effect size) and 1 (large effect size) for binary outcomes. In each setting, we considered *ω*=0 (no covariate effects) and *ω*=1 (with covariate effects).

#### Determining appropriate *c*_max_

To determine the appropriate *c*_max_ for POST, we conducted 100 replications under each of the 5 causal scenarios in simulations A and B and used the decision rule that an OTU is selected as important if *p*-value <0.05 and as not important otherwise. Using this decision rule, we report the true-positive rate (TPR), false-positive rate (FPR) and a composite measure by taking the harmonic mean of TPR and (1-FPR), which is referred to as the pseudo-*F* score. The TPR was obtained by first computing the fraction of detected causal OTUs among all causal OTUs in each replication and then averaging across the 100 replications; the FPR was obtained by first computing the fraction of detected non-causal OTUs among all non-causal OTUs in each replication and then averaging across the 100 replications; and the pseudo-*F* was obtained by taking the harmonic mean of the TPR and 1 −FPR in each replication and then averaging across the 100 replications.

#### Performance assessment

To examine the validity of POST, we set *β*_*m*_=0 for all OTUs and report the type I error rates and the quantile-quantile (Q-Q) plots of the null *p*-values based on 4000 replications under simulations A and B. To examine the performance of selecting causal OTUs, we report the area under the receiver operating characteristic (ROC) curves (AUC in short) based on the 100 replications under each simulation setting from the 5 causal scenarios. AUC can assess the performance over various selection thresholds and account for both TPR and FPR. The AUC was obtained as follows. (1) In each replication, we computed the FDR-adjusted *p*-values of a method, based on which we then computed the FPR and TPR at varying significance thresholds. (2) We took the average FPR and average TPR across the 100 replications at each of the significant thresholds. (3) We constructed the ROC curve using the average FPRs and TPRs and computed the corresponding AUC. We used the two-stage BH (TSBH) FDR procedure [33] to compute the FDR-adjusted *p*-values for all methods except for TF, which directly gives FDR-adjust *p*-values. TSBH has been shown to yield good performance for correlated *p*-values [34] and is implemented in R function “mt.rawp2adjp” of R package *multtest*.

We compare POST with 7 baseline approaches: (1) TreeFDR (TF) of Xiao et al. [14] as implemented by the function “MicrobiomeSeqTreeFDR” in R package *StructFDR*; (2) single-OTU test (SO), by setting *c*=0 in POST; (3) DESeq2 (DE) of Love et al. [35] as implemented by the function “DESeq” with the default Wald test in the R package *DeSeq2*; (4) ANCOM-BC (AB) of Lin et al. [36] as implemented by the function “ancombc” in the R package *ANCOMBC*; (5) LinDA (LD) of Zhou et al. [37] as implemented in the function “linda” in R package *LinDA*; and (6) Wilcoxon rank-sum test on the proportional data (WR-P) and on the CLR transformed data (WR-R) as implemented in the function “wilcox.test.” The Wilcoxon test is only appropriate for binary outcomes with no covariate effects. We also consider (7) Spearman correlation test on proportional data (SC-P) and on the CLR transformed data (SC-R) as implemented by the function “cor.test” of the *stats* R package. The Spearman test is only appropriate for continuous outcomes with no covariate effects. Among these methods, POST and TF incorporate phylogenetic tree information. POST, SO, LD, WR-R, and SC-R use the CLR transformation to address the compositional nature of microbiome data.

## Results

### Simulation results

#### Appropriate *c*_max_

To identify the appropriate value of *c*_max_, the upper bound of *c*, we examined the trajectory of pseudo-*F* (Additional file 1: Fig. S1) over different values of *c*_max_, ranging from 0 (which corresponds to zero OTU neighbors) and 0.1 (which corresponds to a relatively large number of OTU neighbors) as shown in Fig. 2. An ideal *c*_max_ should permit information borrowing only from OTUs within a localized neighborhood in the phylogenetic tree. In addition, because incorporating too much information from irrelevant phylogenetic neighboring OTUs would lead to undesirable TPRs and FPRs, the ideal *c*_max_ is expected to occur at some turning point with which the increment of pseudo-*F* trajectory stops or slows down. Factoring in these two criteria, we found that *c*_max_=0.05 appears to offer a robust choice across the different scenarios in simulations A and B and implemented POST with *c*_max_=0.05 hereafter.

#### Validity of POST

We checked the behavior of POST under the global null hypothesis of no causal OTUs. Table 1 shows that the type I error rate is appropriately controlled at nominal levels of 0.05, 0.01, and 0.001. Additional file 2: Fig. S2 shows the corresponding Q-Q plots of POST *p*-values, where the observed POST *p*-values agree with the expected *p*-values from Uniform (0,1), confirming the validity of POST.

**Table 1:** Type I error rates at the significance levels of 0.05, 0.01, and 0.001 for POST

Simulation	Outcome	*a* = 0.05	*a* = 0.01	*a* = 0.001	*a* = 0.0001
A	Binary	0.047	0.007	0.0006	0.00005
B	Continuous	0.051	0.010	0.0010	0.00008
	Binary	0.047	0.008	0.0006	0.00007

#### Selection performance

**Fig. 4:**
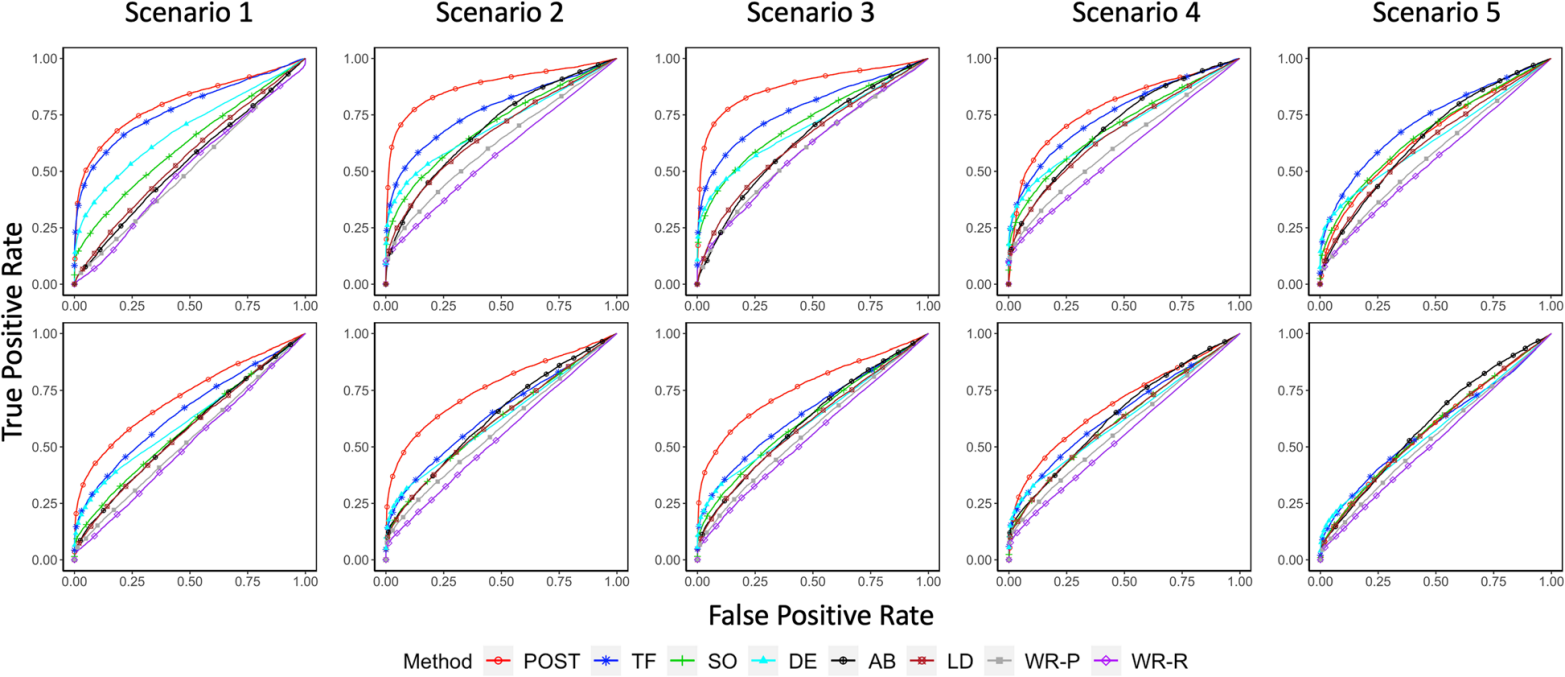
ROC curves of simulation A with large OTU effect size (top row) and small OTU effect size (bottom row) for POST, Single-OTU test (SO), TreeFDR (TF), DESeq2 (DE), ANCOM-BC (AB), LinDA (DA), and Wilcoxon rank-sum test (WR) under the 5 causal scenarios. Scenarios 1 to 3 consider larger “causal hubs,” each containing about 7–10 causal OTUs; scenario 4 considers smaller causal hubs of 2–3 causal OTUs; scenario 5 considers causal OTUs with random positions in the phylogenetic tree

Next, we examined the relative performance of POST against the baseline methods using AUC (Table 2 for simulations A and B without covariate effects; Additional file 3: Table S1 for simulation B with covariate effects). In simulation A, we observe that POST and TF have larger AUC than those methods that do not use tree information when causal OTUs are clustered in large hubs (scenarios 1–3) or small hubs (scenario 4) (Table 2; Fig. 4). Furthermore, POST has larger AUC than TF, suggesting that POST can further boost power through adaptive information collapsing from evolutionarily close OTUs at the level of OTU abundances, compared to TF, which collapses information at the level of OTU *p*-values. The superior performance of POST is consistently observed regardless of whether the effects of nearby causal OTUs are in the same direction (scenarios 1 and 2) or opposite directions (scenarios 3 and 4), and whether the effect sizes are large or small. In scenario 5, where causal OTUs are randomly distributed in the phylogenetic tree, POST performed comparably to the best-performing methods. Among the non-tree based methods, SO, DE, AB, and LD had similar AUCs and are better than the Wilcoxon rank-sum test. The Wilcoxon rank-sum test using proportional data have slightly higher AUC than using CLR transformed data.

**Table 2:** AUC of different methods in simulations A and B. Methods considered include POST, TreeFDR (TF), Single-OTU test (SO) implemented by POST with *c*=0, DESeq2 (DE), ANCOM-BC (AB) and LinDA (LD), Wilcoxon rank-sum test with proportional data (WR-P) or CLR transformed data (WR-R) for binary outcomes, and Spearman correlation test with proportional data (SC-P) or CLR transformed data (SC-R) for continuous outcomes. The outcome values are simulated assuming no covariate effects and 5 different causal OTU scenarios. Scenarios 1 to 3 consider larger “causal hubs,” each containing about 7–10 causal OTUs; scenario 4 considers smaller causal hubs of 2–3 causal OTUs; scenario 5 considers causal OTUs with random positions in the phylogenetic tree. Methods with the highest AUC are shown in bold

Simulation	Simulation A								Simulation B															
Outcome	Binary outcome								Continuous outcome								Binary outcome							
Method	POST	TF	SO	DE	AB	LD	WR-P	WR-R	POST	TF	SO	DE	AB	LD	SC-P	SC-R	POST	TF	SO	DE	AB	LD	WR-P	WR-R
Large effect size*																								
Scenario1	**0.80**	0.78	0.62	0.69	0.54	0.56	0.51	0.51	**0.64**	0.52	0.58	0.61	0.57	0.61	0.57	0.61	0.58	0.53	0.55	0.52	0.57	**0.59**	0.54	0.55
Scenario2	**0.88**	0.77	0.71	0.71	0.70	0.67	0.62	0.58	**0.66**	0.50	0.62	0.65	0.62	0.62	0.57	0.51	0.60	0.48	0.56	0.57	**0.61**	0.56	0.52	0.51
Scenario3	**0.88**	0.78	0.72	0.70	0.64	0.64	0.60	0.60	**0.70**	0.49	0.62	0.63	0.61	0.62	0.58	0.51	**0.63**	0.50	0.59	0.55	0.58	0.58	0.57	0.51
Scenario4	**0.78**	0.75	0.70	0.69	0.70	0.67	0.62	0.58	**0.63**	0.48	0.61	**0.63**	0.58	0.61	0.58	0.52	**0.56**	0.49	**0.56**	0.54	0.55	0.55	0.55	0.51
Scenario5	0.66	**0.72**	0.67	0.64	0.65	0.63	0.57	0.55	0.59	0.48	0.60	**0.61**	0.59	0.60	0.54	0.51	0.54	0.50	0.54	0.52	**0.57**	0.55	0.53	0.50
Small effect size*																								
Scenario1	**0.72**	0.65	0.59	0.62	0.57	0.57	0.53	0.51	**0.62**	0.48	0.56	0.58	0.56	0.59	0.55	0.56	0.56	0.49	0.53	0.52	0.54	**0.57**	0.52	0.52
Scenario2	**0.76**	0.65	0.61	0.62	0.63	0.62	0.58	0.55	**0.62**	0.49	0.59	0.61	0.59	0.59	0.54	0.51	0.54	0.49	0.53	0.55	**0.56**	0.53	0.51	0.50
Scenario3	**0.76**	0.65	0.63	0.62	0.62	0.60	0.57	0.55	**0.66**	0.49	0.60	0.60	0.58	0.60	0.56	0.50	**0.59**	0.50	0.56	0.55	0.55	0.56	0.54	0.50
Scenario4	**0.69**	0.65	0.62	0.62	0.64	0.62	0.58	0.55	**0.60**	0.48	0.58	**0.60**	0.57	0.58	0.56	0.51	**0.54**	0.50	0.53	0.53	0.52	**0.54**	0.53	0.51
Scenario5	0.59	0.58	0.59	0.58	**0.60**	0.58	0.54	0.52	0.56	0.50	0.56	**0.57**	0.56	**0.57**	0.53	0.50	**0.53**	0.50	0.52	0.52	**0.53**	**0.53**	0.52	0.50

*For simulation A, small OTU effect size is from *N*(±1,1) and large OTU effect size is from *N*(±2,1). For simulations B, small OTU effect size is simulated from *N*(±0.2, 0.04) and *N*(±0.3,0.06) for continuous and binary outcomes, respectively; large OTU effect size is simulated from *N*(±0.5, 0.1) and *N*(±1,0.2) for continuous and binary outcomes, respectively

In simulation B, POST has the largest AUC or similar AUC to the best-performing method in all scenarios, regardless of the effect sizes, without covariates (Table 2) or with covariates (Additional File 3: Table S1). DE, AB, and LD had similar AUCs and can perform the best even in some of scenarios 1–4 although they did not incorporate tree information. We also observe that TF has an AUC around 0.5 in most scenarios. A closer examination suggests that this may be because the signal strengths in simulation B are much smaller than those in simulation A, based on the way that the data were simulated. Consequently, TF yields many large, tied FDR adjusted *p*-values in simulation B, while other methods yield fewer tied adjusted *p*-values with relatively smaller values than those of TF.

In summary, across different simulation scenarios, POST has better or comparable performance compared to the baseline methods. POST tends to have the largest AUC among all methods (e.g., in scenarios 1–4 of informative trees); if not (e.g., in scenario 5 of non-informative trees), POST has its AUC comparable to the top methods, which can either be TF, SO, DE, AB or LD, depending on simulation settings.

### Real data analysis

We applied POST to two microbiome datasets, both at the level of OTUs formed at ≤3% dissimilarity and at the level of ASVs. We present the OTU-level results below and the ASV-level results in Additional file 7: Section S1. In all analyses, we considered TF, SO, AB, LD, WR-P, and WR-R as baselines. We included race as a covariate except for WR-based methods. We used the TSBH procedure [33] to compute the FDR-adjusted *p*-values except for TF, which outputted its own FDR-adjusted *p*-values. We selected important OTUs/ASVs using the decision rule of FDR-adjusted *p*-values <0.05.

#### Association study of vaginal microbiome and bacterial vaginosis

Bacterial vaginosis (BV) is a type of vaginal inflammation and is characterized by low levels of lactobacilli and overgrowth of various anaerobic bacteria [38]. BV is most often diagnosed using the Amsel criteria or microscopy-based Nugent scoring [39] but with limited accuracy [40]. High-throughput sequencing technologies such as 16S rRNA amplicon sequencing have been used to study the species diversity of vaginal microbiota and have shown promise as an alternative assessment of BV for practical and accurate diagnosis [41–43].

We conducted an OTU-specific analysis using the 16S rRNA gene sequencing dataset of vaginal microbiome from [43] to evaluate the association between BV and the normal vaginal microbiome. The dataset consists 39 individuals, and the sequencing data and metadata are publicly available at NCBI SRA database (PRJNA600021). Initial processing leads to the abundance data of 2711 OTUs formed at 97% similarity. We employed *FastTree2* [23] to infer the phylogenetic tree using the OTU sequences. Finally, we filtered out OTUs with abundance < 0.005% and prevalence < 10% and analyzed the resulting 186 OTUs of 39 individuals for BV association studies.

**Fig. 5:**
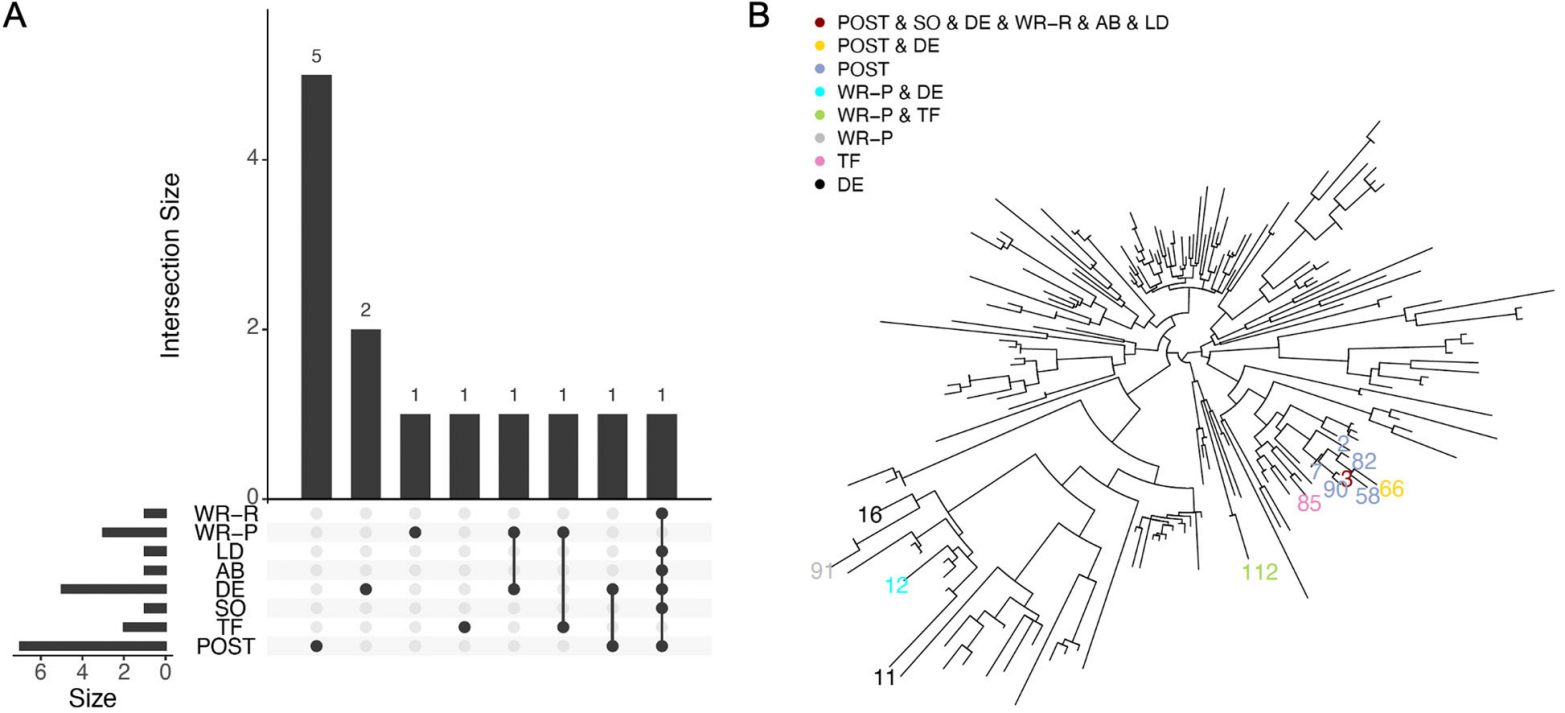
Upset plot of detected OTUs at FDR level of 0.05 and phylogenetic trees of the analyzed OTUs with detected OTUs for bacterial vaginosis study. SO, single-OTU test implemented by POST with *c* = 0; TF, TreeFDR; DE, DESeq2; AB, ANCOM-BC; LD, LinDA; WR-P, Wilcoxon rank-sum test using proportional data; WR-R, Wilcoxon rank-sum test using CLR transformed data

The Upset plot (Fig. 5A) shows that POST, TF, SO, DE, AB, LD, WR-P, and WR-R identified 7, 2, 1, 5, 1, 1, 3, and 1 significant OTUs, respectively. Table 3 lists the OTUs detected by each method and their mapped genus/species with 100% sequence identity among vaginal microbiome. We organized our findings as follows. (i) Among the 7 POST-detected OTUs, OTU3 (mapped to *Lactobacillus crispatus*) is also identified by SO, DE, AB, LD, and WR-R. Depletion of *Lactobacillus crispatus* has been shown to be highly associated with BV [44]. POST also identifies 6 additional OTUs as important (among which OTU66 is also identified by DE); these OTUs are all mapped to *Lactobacillus*species, including *Lactobacillus jensennii*, *Lactobacillus gasseri*, and *Lactobacillus iners*. These vaginal *Lactobacillus* species have been shown to have different abundances in women with vs. without BV [44] and play important roles in ithe vaginal ecosystem [45]. (ii) TF identified 2 OTUs, i.e., OTU112 (mapped to *Peptoniphilus* sp. and also identified by WR-P) and OTU85 (mapped to *Gemella* sp.); these genera have also been detected in BV cases [46, 47]. (iii) Besides the OTUs discussed above, DE and/or WR-P also identify 4 other OTUs (i.e., OTU11, OTU12, OTU16, and OTU91), which are all mapped to genus *Prevotella*, and OTU16 (*Prevotella timonensis*) has been found to be associated with BV [48]. (iv) It seems that POST, SO, LD, and WR-R (i.e., methods based on CLR-transformed data) miss some of the OTUs identified by TF, DE, and WR-P (i.e., methods based on proportional data), although there are also OTUs detected by both types of methods. (v) Fig. 5B shows the relative positions of the identified OTUs in the phylogenetic tree, where POST identified OTUs tend to be more clustered together compared to other methods.

**Table 3:** OTUs significantly associated with bacterial vaginosis (BV) at FDR level of 0.05. TF, TreeFDR; SO, Single-OTU test implemented by POST with *c* = 0; DE, DESeq2; AB, ANCOM-BC; LD, LinDA; WR-P, Wilcoxon rank-sum test using proportional data; WR-R, Wilcoxon rank-sum test using the CLR transformed data

OTU	FDR-adjusted *p*-value								Detected method	Genus/species	Direction**
	POST	TF	SO	DE	AB	LD	WR-P*	WR-R*			
OTU3	0.039	0.089	0.045	0.002	0.006	0.041	0.178	0.025	POST/SO/DE/AB/LD/WR-R	*Lactobacillus crispatus*	-
OTU90	0.039	0.245	0.953	0.950	0.983	0.961	0.375	0.989	POST	*Lactobacillus* sp.	+
OTU7	0.039	0.653	0.881	0.147	0.766	0.729	0.760	0.932	POST	*Lactobacillus jensenii*	-
OTU82	0.039	0.517	0.985	0.970	0.963	0.976	0.258	0.989	POST	*Lactobacillus gasseri*	-
OTU66	0.039	0.544	0.701	0.027	0.379	0.615	0.378	0.932	POST/DE	*Lactobacillus* sp.	-
OTU2	0.039	0.180	0.917	0.876	0.766	0.923	0.235	0.989	POST	*Lactobacillus iners*	+
OTU58	0.039	0.839	0.917	0.876	0.871	0.923	0.791	0.989	POST	*Lactobacillus* sp.	+
OTU112	0.411	0.046	0.701	0.422	0.379	0.678	0.040	0.932	TF/WR-P	*Peptoniphilus* sp.	+
OTU85	0.470	0.046	0.906	0.372	0.766	0.870	0.097	0.989	TF	*Gemella* sp.	+
OTU11	0.918	0.286	0.943	0.000	0.869	0.923	0.220	0.989	DE	*Prevotella* sp.	+
OTU12	0.391	0.089	0.701	0.013	0.338	0.615	0.040	0.942	DE/WR-P	*Prevotella* sp.	+
OTU16	0.680	0.155	0.881	0.001	0.766	0.918	0.097	0.989	DE	*Prevotella timonensis*	+
OTU91	0.680	0.092	0.881	0.505	0.766	0.852	0.040	0.989	WR-P	*Prevotella* sp.	+

*WR-P and WR-R did not adjust for race

**+ (and −) indicates that the OTU is positively (and negatively) associated with BV risk from a logistic regression

We further illustrated the data-adaptiveness of POST using OTU2 (average proportional abundance 0.22, mapped to *Lactobacillus iners*, and only identified by POST) and OTU3 (average proportional abundance 0.26, mapped to *Lactobacillus crispatus*, and identified by POST, SO, DE, AB, LD, and WR-R). The abundance boxplots (Additional file 4: Fig. S3) show that OTU2 has higher abundance in BV patients compared to healthy women, while OTU3 has an opposite pattern. Our findings agree with the literature that BV patients have loss of many *Lactobacillus* species except *Lactobacillus iners* [44, 49]. When testing for OTU2, the best *c* determined by the data is *c*=0.03, suggesting some information borrowing from neighboring OTUs (Additional file 5: Fig. S4A), e.g., OTU7 has *r*_7,2_=0.74 and OTU3 has *r*_3,2_=0.52 in Eq. (2). This example illustrates that (i) POST can incorporate information with opposite directions, and (ii) the information borrowing is OTU-specific and data-driven—when testing for OTU3, the best *c* is *c*=0, i.e., no information borrowing from any neighboring OTUs (Additional file 5: Fig. S4B).

#### Association study of vaginal microbiome and preterm birth

Preterm birth is a major cause of neonatal morbidity and mortality. Previous studies have suggested that the risk of preterm birth is associated with vaginal microbiota composition, especially certain species/genera including *Lactobacillus crispatus*, *Lactobacillus iners*, *BVAB1*, *Sneathia amnii*, and some *Prevotella* species [12, 20, 50]. We performed an OTU-specific analysis on the Stanford cohort data of Callahan et al. [50] to identify OTUs associated with preterm birth. We applied the same data processing steps as in the BV study and obtained 746 OTUs and 39 individuals. After excluding OTUs with abundance <0.005*%* and present rate <10*%*, we based our analysis on the 95 remaining OTUs.

**Fig. 6:**
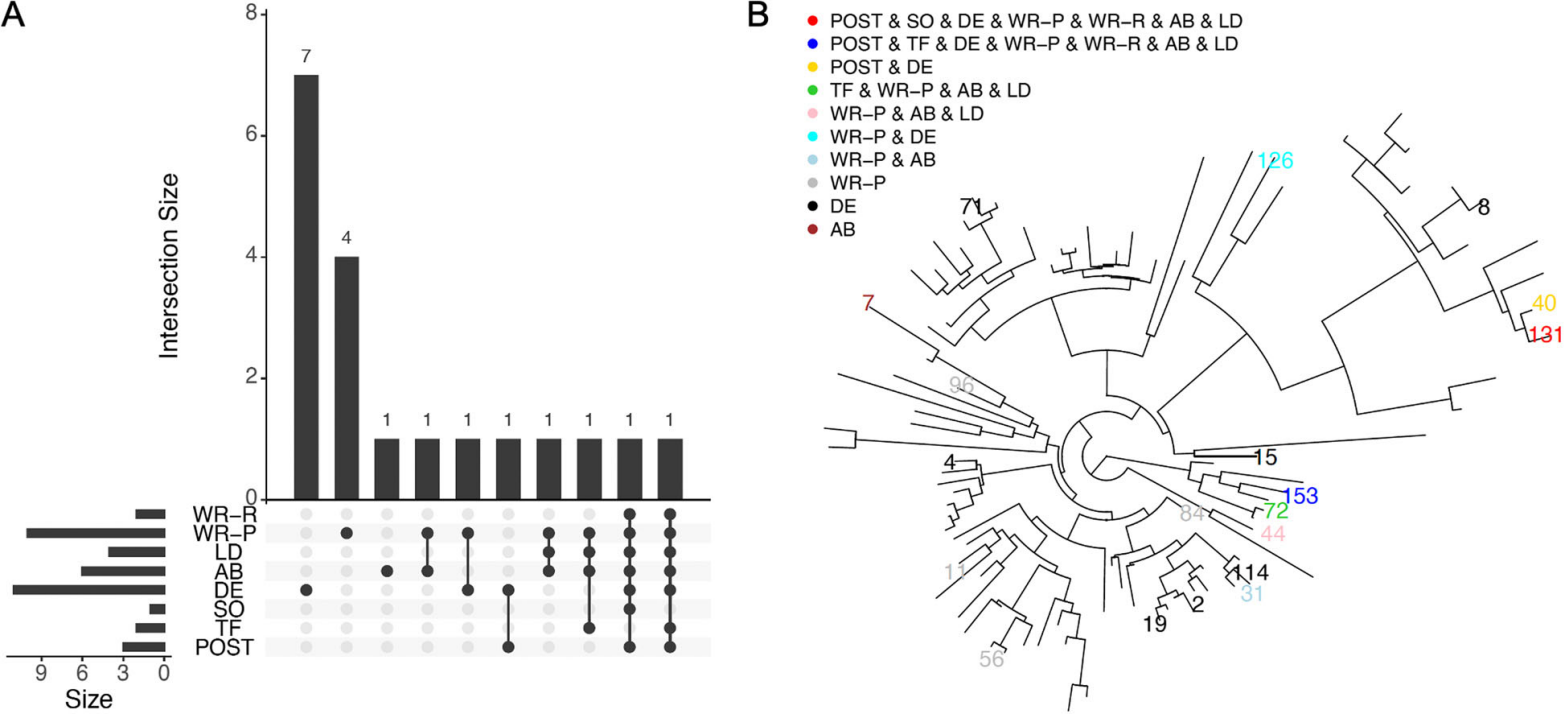
Upset plot of detected OTUs at FDR level of 0.05 and phylogenetic trees of the analyzed OTUs with detected OTUs for preterm birth study. SO, Single-OTU test implemented by POST with *c* = 0; TF, TreeFDR; DE, DESeq2; AB, ANCOM-BC; LD, LinDA; WR-P, Wilcoxon rank-sum test using proportional data; WR-R,Wilcoxon rank-sum test using the CLR transformed data

Figure 6A shows that POST, TF, SO, DE, AB, LD, WR-P, and WR-R identified 3, 2, 1, 11, 6, 4, 10, and 2 significant OTUs, respectively. Additional file 6: Table S2 shows the significant OTUs, their detection methods, and the corresponding genus/species. Figure 6B shows the relative positions of the identified OTUs in the phylogenetic tree. The results can be summarized as follows. (i) All of the 3 POST-detected OTUs have also been detected by at least 1 baseline method, i.e., OTU131 (mapped to *Prevotella* sp.), which is also found significant by all baseline methods except TF; OTU40 (mapped to *Prevotella melaninogenica*), which is also identified by DE; and OTU153 (mapped to *Neisseria* sp.), which is also identified by all baseline methods except SO. The *Prevotella* genus has been reported to be associated with preterm birth [12]; OTU153 has 97.45% (229/235) sequence identity with *Neisseria gonorrhoeae*, which causes one type of sexually transmitted infection and has been reported to be associated with preterm birth [51, 52]. (ii) There were 4 OTUs that were identified by ≥1 baseline method but missed by POST: OTU72, OTU126, OTU31, and OTU44. OTU72 (mapped to *Haemophilus parainfluenzae*) was identified by TF, AB, LD, and WR-P, and *Haemophilus parainfluenzae* has been reported to play a potential role in preterm birth [53]. OTU44 (mapped to *Fusobacterium nucleatum* and identified by AB, LD, and WR-P), has been reported associated with preterm birth [54]. We found no supporting evidence in the literature regarding the association with preterm birth for OTU126 (mapped to *Cloacibacterium* sp. and identified by DE and WR-P) or for OTU31 (mapped to *Staphylococcus anginosus* and identified by AB and WR-P).

(iii) Besides the OTUs discussed above, DE uniquely identified 7 OTUs. Among these OTUs, OTU2 (mapped to *Lactobacillus crispatus*) and OTU8 (mapped to *Prevotella* sp.) have been found associated with preterm birth in the literature [12, 20]. OTU4 (mapped to Veillonellaceae bacterium), OTU71 (mapped to *Alloscardovia omnicolens*), and OTU19 (mapped to *Staphylococcus aureus*) have also be discussed in the literature, although they are reported as not associated with preterm birth [55, 56]. We found no supporting evidence in the literature regarding the association with preterm birth for OTU114 (mapped to *Lacticaseibacillus rhamnosus*) or for OTU15 (mapped to *Mycoplasma hominis*). (iv) AB uniquely identified OTU7 (mapped to *Clostridiales genomosp. BVAB1*), which has been reported to be associated with preterm birth [12]. (v) WR-P uniquely identified 4 OTUs, for which we did not find supporting evidence in the literature, and the associations diminish if switching to WR-R. (vi) We also observed that the CLR-based methods (e.g., POST, SO, LD, and WR-R) tend to identify fewer OTUs compared to the proportional-based methods (e.g., DE and WR-P).

A final note is that the two data applications show different relative performance of POST compared to the baseline methods. In the BV study, POST detected several significant unique OTUs that were not identified by other methods. We discussed the biological relevance of these identified OTUs and examined the phylogenetic relationship among the identified OTUs (Fig. 5B). The results suggest that these OTUs tend to be evolutionarily close and their signals might be easier to be detected by borrowing an appropriate amount information from appropriate neighboring OTUs. In contrast, in the preterm birth study, POST only identified OTUs that are also identified by other non-tree-based methods. In particular, POST had similar performance to SO, LD, and WR-R, suggesting the compatibility of POST to the non-tree based methods on CLR transformed data and the robustness of POST to non-informative phylogeny.

## Discussion and conclusions

In this work, we proposed a phylogeny-guided OTU-specific test, POST. POST extends the kernel-based approaches from community-level analysis to OTU-level analysis, and evaluates the association of a focal OTU based on the abundance of itself as well as its phylogenetically close neighbors. POST allows closer neighboring OTUs to contribute more than distant neighbors and includes the standard single OTU tests as a special case (i.e., zero contribution from all neighboring OTUs). The actual contribution from neighboring OTUs is adaptively determined by data, using a tuning parameter *c* to identify the appropriate OTUs and the optimal amount from which to borrow information. We evaluated the performance of POST in different simulated scenarios and in real data applications. We showed that POST can enhance the selection performance by yielding more desirable true positive and false-positive detection when compared to commonly used tree-free and tree-guided methods: POST tends to have higher AUC when the phylogenetic tree is informative and have similar AUC when the phylogenetic tree is not informative.

Here, we described POST based on OTUs formed at ≤3% sequence dissimilarity and the corresponding phylogenetic tree constructed using *FastTree2* [23]. Although our specific testing results are restricted to this framework, the POST method is relevant to a broader class of metagenomic sequencing methods including marker-gene and shotgun sequencing methods, where reads are assigned taxonomy against a reference database with a pre-calculated phylogeny. However, there are a couple of points to keep in mind when extending the described framework to these more general scenarios. Recall that for a target OTU, its neighborhood and the amount of information to borrow are controlled via *c*. Because *c* is measured on the scale of the SD of pairwise OTU distances in the phylogenetic tree, *c* does not depend on the actual values of the OTU distance in a tree, and thus, POST can be applied on phylogenetic trees from different tools. For different taxonomic levels, some modifications may be needed because the upper bound of *c*, *c*_max_, which controls the neighborhood boundary of an OTU, may or may not always be generalizable to OTUs defined at different level. For example, it would be dangerous to directly apply the current *c*_max_ to OTUs formed at > 3% dissimilarity or higher taxonomic ranks (e.g., genus) because the current *c*_max_ may lead to too large a neighborhood in the tree for the focal OTU. On the other hand, the current *c*_max_ can be used for OTUs formed at ASV levels (i.e., 0% dissimilarity), with the price of possibly having a smaller OTU neighborhood than the actual optimal neighborhood to borrow information from. When needed, the proper *c*_max_ can be explored and determined using a similar simulation procedure as conducted in the paper so to assure that only OTUs from a meaningful, localized neighborhood would contribute to the association assessment of the focal OTU.

It is possible to extend the POST framework to incorporate OTU correlation based on abundance/co-occurrence instead of phylogeny. Method 1 is to replace the OTU phylogenetic correlation (*r*_*ℓm*_) in step 1 with the OTU abundance correlations (such as computing *r*_*ℓm*_ using SparCC [57]). A hyperparameter c will need to be integrated into *r*_*ℓm*_ to permit that the information borrowing is conducted in an outcome-supervised fashion. Method 2 is to replace the input phylogenetic tree in step 1 with a tree constructed based on abundance correlation or some network structures [58], e.g., by first converting the abundance correlation to a dissimilarity matrix and then performing hierarchical clustering to form a “tree” as outlined in Bichat et al. [19]. Method 2 can be directly implemented by the existing POST R package *POSTm*, although additional numerical studies would be needed to determine an appropriate *c*_max_ value. However, method 2 appears to incorporate some redundancy because in the existing POST workflow, the “tree” will then be converted into correlation (i.e., *r*_*ℓm*_). Finally, given that which correlation type (abundance vs. phylogeny) would be informative of the underlying OTU association patterns is unknown in real practice, an important next step will be to construct an omnibus framework that can adaptively determine “which types” of correlation information to use besides “how much” information to borrow from correlated OTUs.

In the real data analyses, we added a pseudo-count of 0.5 to the OTU count tables to handle the zero-count issues. It has been shown in the literature that the choice of pseudo-count can affect the association results [59, 60]. For POST, we conducted sensitivity analyses to assess the impact of pseudo-counts, by comparing the results of pseudo-count 0.5 and pseudo-count 1 under simulation A (Additional file 8: Table S3) and in real data analyses (Additional file 9: Fig. S5). We observed that (1) the AUCs based on different pseudo-counts (Additional file 8: Table S3) are very similar across different scenarios and effect sizes. (2) The *p*-values obtained using different pseudo-counts (Additional file 9: Fig. S5) are highly correlated and fall along the 45-degree lines in the scatter plots. While some deviations may occur (e.g., in the BV analysis), the deviations were from non-small *p*-values. These results suggest that POST is fairly robust to the choices of pseudo-counts.

In POST, we build the kernel matrix based on the non-phylogenetic Aitchison distance to account for the compositional nature of microbiome data [61]. It is possible to construct local kernel using other distance metrics, such as the UniFrac distance family. For example, we can compute the local weighted UniFrac (WU) distance for OTU *m* between subjects *i* and *j* by $\boldsymbol {WU}_{ij}^{m} = \frac {\sum _{t=1}^{T} b_{t}|[\boldsymbol {r}_{m}]_{t}^{\top }(\boldsymbol {q}_{t}^{i}-\boldsymbol {q}_{t}^{j})|}{\sum _{t=1}^{T} b_{t}|[\boldsymbol {r}_{m}]_{t}^{\top }(\boldsymbol {q}_{t}^{i}+\boldsymbol {q}_{t}^{j})|}$ where *b*_*t*_ is the length of branch *t* and *T* is the total number of branches. Assuming that there are *n*_*t*_ OTUs connected to branch *t* in the phylogenetic tree, then $\boldsymbol {q}_{t}^{i}$ is a length- *n*_*t*_ vector recording the abundance proportions of the *n*_*t*_ OTUs connected to branch *t* for subject *i*; [***r***_*m*_]_*t*_ is a length- *n*_*t*_ vector recording the correlation between OTU *m* and those OTUs connected to branch *t* as obtained in the “Step 1: Quantifying OTU phylogenetic correlation” section and dependent on *c*. The above equation quantifies the “local” weighted UniFrac distance for OTU *m* by weighting the proportion difference of the branch-*t*-related OTUs according to their correlations with OTU *m*. If ***r***_*m*_ equals ***1***, the above equation is the global weighted UniFrac distance. However, in these phylogenetic distances, the phylogenetic information seems to be used twice, one embedded in the original distance definition and one in the use of ***r***_*m*_. Further examinations are needed to understand the pros and cons of local tests on phylogenetic-based distance.

## Supplementary Information

**Additional file 1** Figure S1. Pseudo-F plots from Simulations A and B with large effect size (left) and small effect size (right) of causal OTUs for all five scenarios. Simulation A considers binary outcomes (1st row), which were simulated assuming no covariate effects besides OTU effects; Simulations B consider both binary outcomes (2nd rows) and continuous outcomes (3rd rows), which were simulated assuming OTU and covariate effects.

**Additional file 2** Figure S2. QQ plot of POST *p*-values under the null hypothesis of no causal OTUs. The plots from left to right are for Simulations A, B-continuous and B-binary respectively. The outcomes in Simulation A were generated assuming no OTU effects and no covariate effects; the outcomes in Simulations B were generated assuming no OTU effects but with covariate effects.

**Additional file 3** Table S1. AUC of different methods in Simulation B. The methods considered include POST, TreeFDR (TF), Single-OTU test (SO), DESeq2 (DE), ANCOM-BC (AB) and LinDA (LD). The outcome values are simulated assuming covariate effects and 5 different causal-OTU scenarios. Scenarios 1 to 3 consider larger “causal hubs”, each containing about 7–10 causal OTUs; Scenario 4 considers smaller causal hubs of 2–3 causal OTUs; Scenario 5 considers causal OTUs with random positions in the phylogenetic tree. Wilcoxon rank-sum test (WR; for binary outcomes) and Spearman correlation test (SC; for continuous outcomes) are not included because they cannot account for covariates. Methods with the highest AUC are shown in bold.

**Additional file 4** Figure S3. Boxplots of OTU2 (*Lactobacillus.iners*) and OTU3 (*Lactobacillus.crispatus*) for BV and healthy women in the BV study.

**Additional file 5** Figure S4. OTU phylogenetic correlation for OTU2 and OTU3 with best *c* values 0.03 and 0, respectively. Only neighboring OTUs inside genus *Lactobacillus* are shown.

**Additional file 6** Table S2. OTUs significantly associated with preterm birth at FDR level of 0.05. TF: TreeFDR; SO: Single-OTU test implemented by POST with c=0; DE: DESeq2; WR-P: Wilcoxon rank-sum test using proportional data; WR-R: Wilcoxon rank-sum test using CLR transformed data.

**Additional file 7** Section S1. In this section, we conducted association analysis at the ASV level (i.e., 0% dissimilarity) using the same analysis strategies as described in the main texts for the bacterial vaginosis (BV) and the preterm birth (PTB) association studies.

**Additional file 8** Table S3. The AUC using different pseudo-counts in Simulation A. The AUCs based on pseudo-count 0.5 are very close to the AUCs based on pseudo-count 1 across different scenarios and effect sizes.

**Additional file 9** Figure S5. Scatter plots of -log10 *p*-values obtained using pseudo-count 0.5 (X-axis) and those obtained using pseudo-count 1 (Y-axis) in the BV study (left) and the PTB study (right). The *p*-values of different pseudo-counts are highly correlated (R=0.995 in BV and 0.998 in PTB) and fall along the 45-degree lines.

## Data Availability

The dataset of upper respiratory tract microbiome study [24] used in our simulation study is available in R package *GUniFrac*. The 16S rRNA sequencing dataset and metadata of the vaginal microbiome and bacterial vaginosis study [43] used in the real data analysis are publicly available at NCBI SRA database with project number PRJNA600021. The processed sequencing data and metadata of the vaginal microbiome and preterm birth [50] used in the real data analysis are publicly available at the Stanford Digital Repository (https://purl.stanford.edu/yb681vm1809). Our POST method is implemented by R package *POSTm*, and it is now available at CRAN (https://CRAN.R-project.org/package=POSTm). The R codes used for simulation, real data analysis, and data visualization are available through GitHub by request.

## Abbreviations

POST: Phylogeny-guided microbiome OTU-specific association test
OTU: Operational taxonomic units
ASV: Amplicon sequence variant
FPR: False-positive rate
TPR: True-positive rate
AUC: Area under the receiver operating characteristic curves
TSBH: Two-stage Benjamini–Hochberg procedure
TF: TreeFDR
DE: DESeq2
AB: ANCOM-BC
LD: LinDA
WR-P: Wilcoxon rank-sum test using proportional data
WR-R: Wilcoxon rank-sum test using CLR transformed data
SO: Single-OTU test
BV: Bacterial vaginosis
WU: weighted UniFrac
