# Supplementary figures and images for: Phylogeny-guided microbiome OTU-specific association test (POST)
Source: Microbiome. 2022 Jun 7;10:86. doi: 10.1186/s40168-022-01266-3 (PMC9171974; doi:10.1186/s40168-022-01266-3)

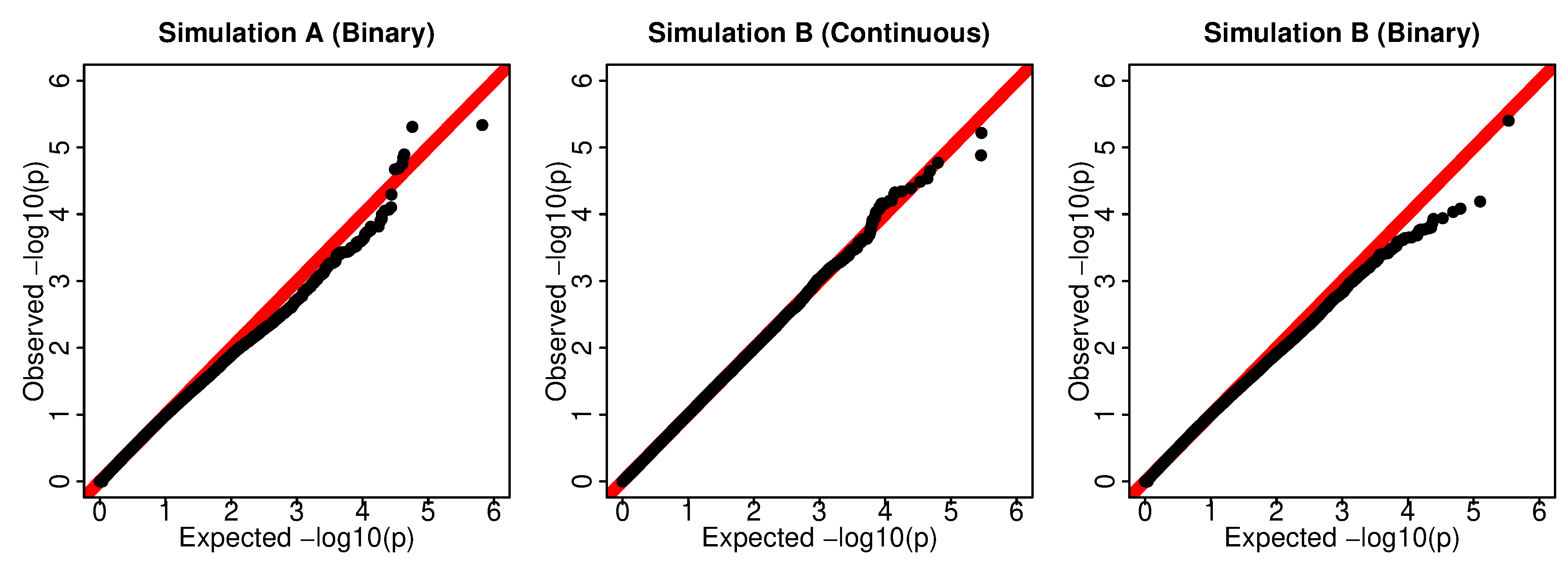

Supplement: Supplementary file 3 — Additional file 2 Figure S2. QQ plot of POST p-values under the null hypothesis of no causal OTUs. The plots from left to right are for Simulations A, B-continuous and B-binary respectively. The outcomes in Simulation A were generated assuming no OTU effects and no covariate effects; the outcomes in Simulations B were generated assuming no OTU effects but with covariate effects. [file 40168_2022_1266_MOESM2_ESM.jpeg]

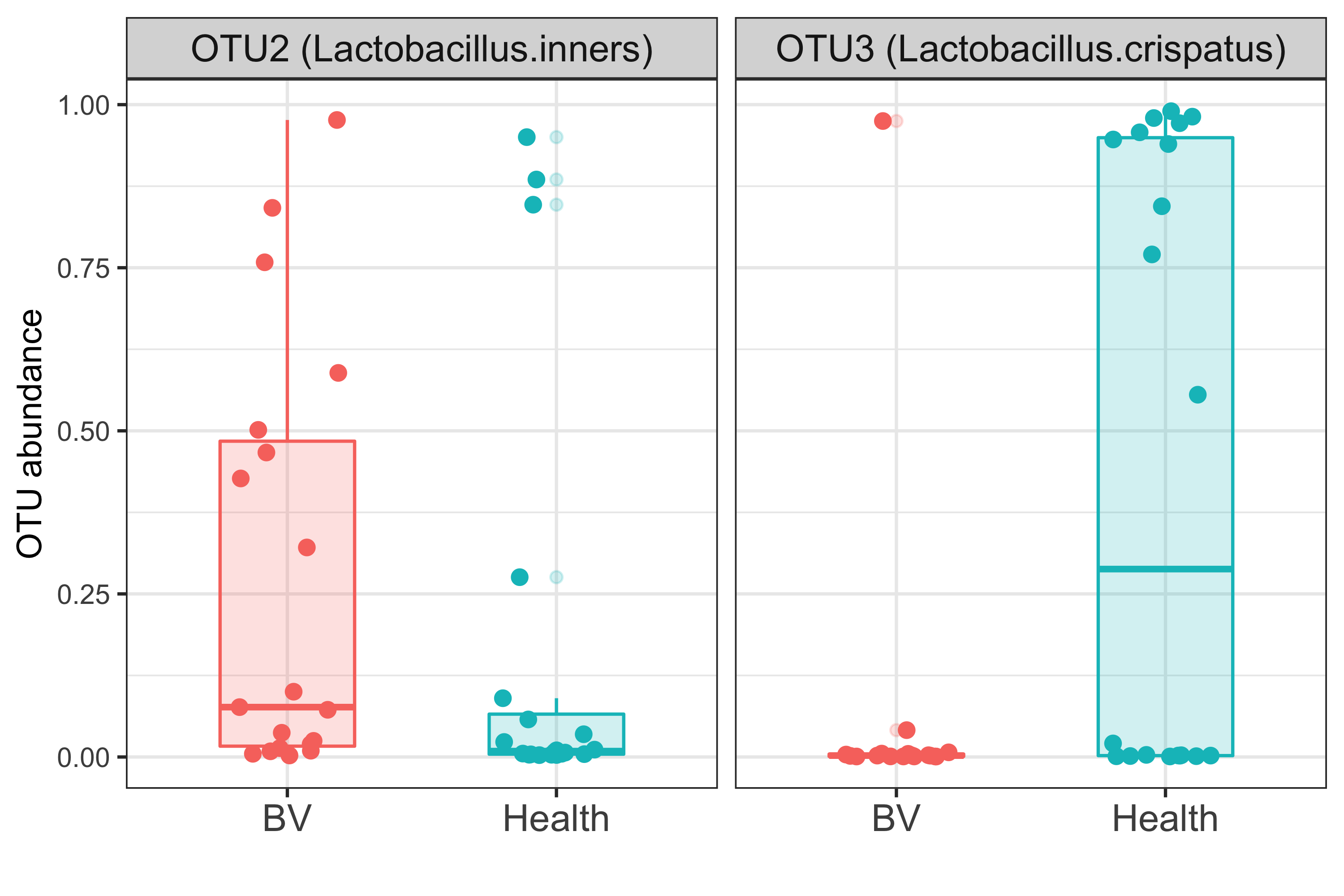

Supplement: Supplementary file 5 — Additional file 4 Figure S3. Boxplots of OTU2 (Lactobacillus.iners) and OTU3 (Lactobacillus.crispatus) for BV and healthy women in the BV study. [file 40168_2022_1266_MOESM4_ESM.png]

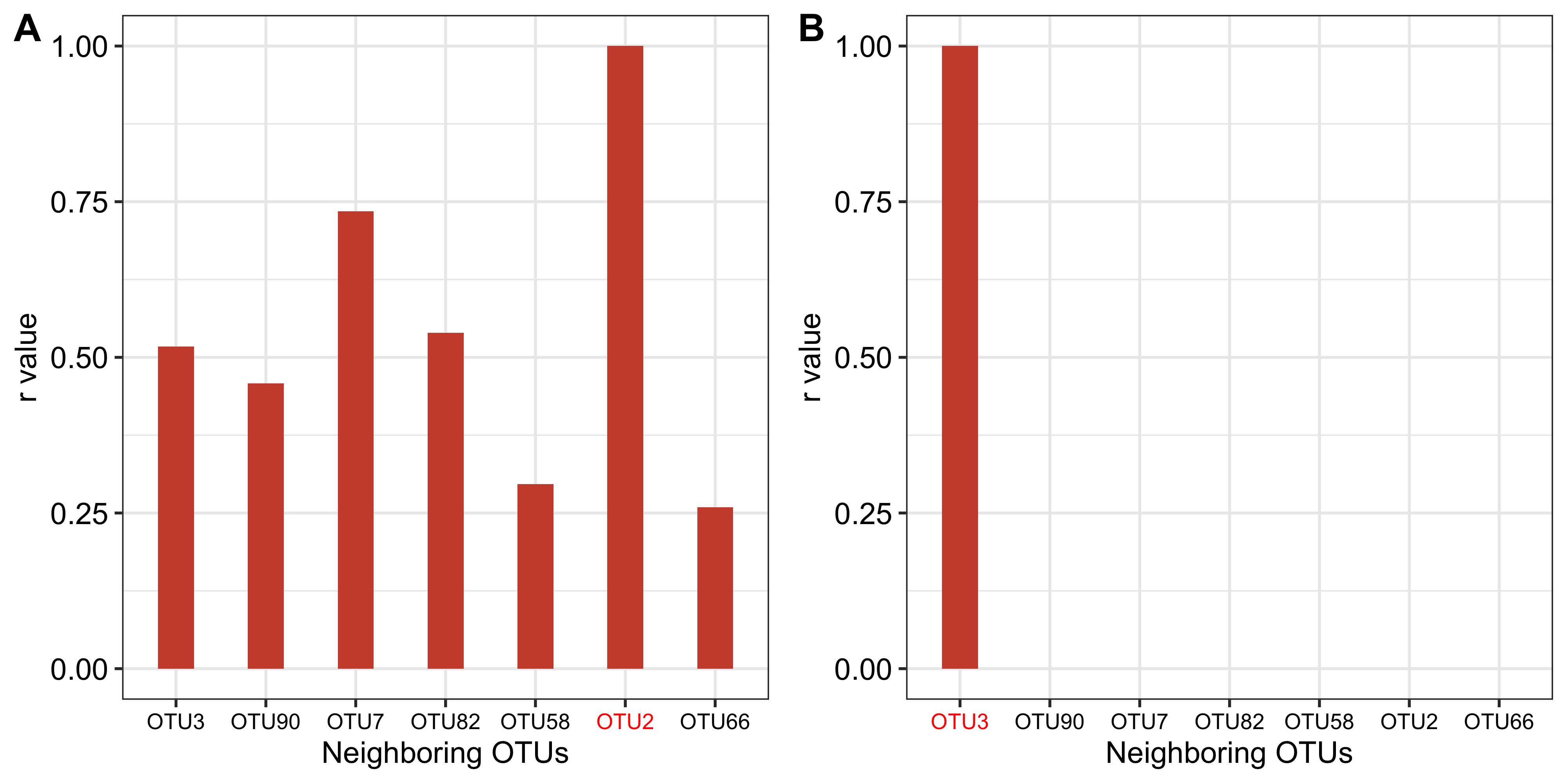

Supplement: Supplementary file 6 — Additional file 5 Figure S4. OTU phylogenetic correlation for OTU2 and OTU3 with best c values 0.03 and 0, respectively. Only neighboring OTUs inside genus Lactobacillus are shown. [file 40168_2022_1266_MOESM5_ESM.png]

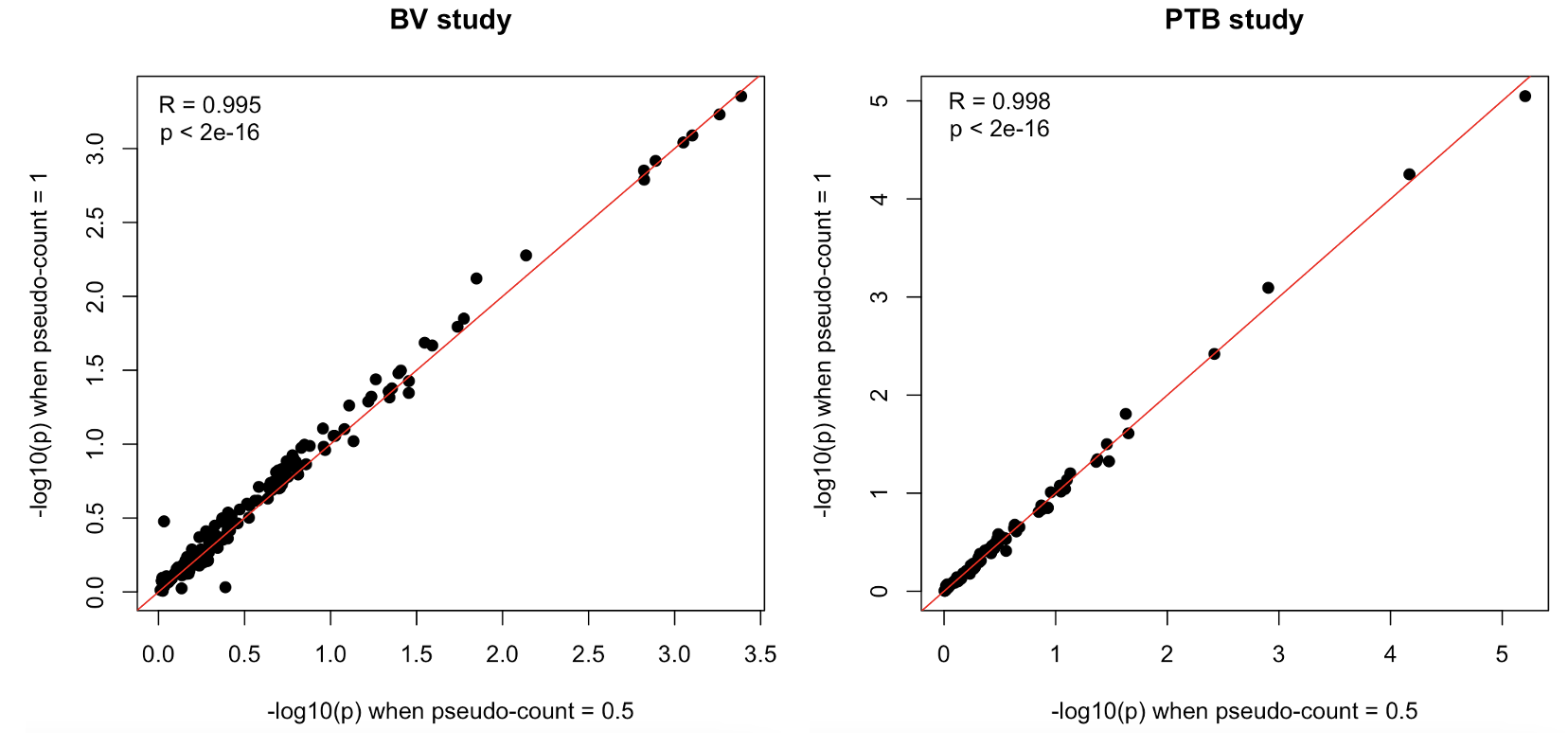

Supplement: Supplementary file 10 — Additional file 9 Figure S5. Scatter plots of -log10 p-values obtained using pseudo-count 0.5 (X-axis) and those obtained using pseudo-count 1 (Y-axis) in the BV study (left) and the PTB study (right). The p-values of different pseudo-counts are highly correlated (R=0.995 in BV and 0.998 in PTB) and fall along the 45-degree lines. [file 40168_2022_1266_MOESM9_ESM.png]
