# Supplementary material for: Phylogeny-guided microbiome OTU-specific association test (POST)
Source: Microbiome. 2022 Jun 7;10:86. doi: 10.1186/s40168-022-01266-3 (PMC9171974; doi:10.1186/s40168-022-01266-3)
Supplement: Supplementary file 4 — Additional file 3 Table S1. AUC of different methods in Simulation B. The methods considered include POST, TreeFDR (TF), Single-OTU test (SO), DESeq2 (DE), ANCOM-BC (AB) and LinDA (LD). The outcome values are simulated assuming covariate effects and 5 different causal-OTU scenarios. Scenarios 1 to 3 consider larger “causal hubs”, each containing about 7–10 causal OTUs; Scenario 4 considers smaller causal hubs of 2–3 causal OTUs; Scenario 5 considers causal OTUs with random positions in the phylogenetic tree. Wilcoxon rank-sum test (WR; for binary outcomes) and Spearman correlation test (SC; for continuous outcomes) are not included because they cannot account for covariates. Methods with the highest AUC are shown in bold. [file 40168_2022_1266_MOESM3_ESM.docx]

**Table S1.** AUC of different methods in Simulations B. The methods considered include POST, TreeFDR (TF), Single-OTU test (SO), DESeq2 (DE), ANCOM-BC (AB) and LinDA (LD). The outcome values are simulated assuming covariate effects and 5 different causal-OTU scenarios. Scenarios 1 to 3 consider larger "causal hubs", each containing about 7-10 causal OTUs; Scenario 4 considers smaller causal hubs of 2-3 causal OTUs; Scenario 5 considers causal OTUs with random positions in the phylogenetic tree. Wilcoxon rank-sum test (WR; for binary outcomes) and Spearman correlation test (SC; for continuous outcomes) are not included because they cannot account for covariates. Methods with the highest AUC are shown in bold.

| Simulation | | Simulation B | | | | | | | | | | | |
| --- | --- | --- | --- | --- | --- | --- | --- | --- | --- | --- | --- | --- | --- |
| Outcome | | Continuous outcome | | | | | | Binary outcome | | | | | |
| Method | | POST | TF | SO | DE | AB | LD | POST | TF | SO | DE | AB | LD |
| Large OTU Effect Size* | Scenario1 | **0.71** | 0.49 | 0.61 | 0.59 | 0.54 | 0.60 | **0.57** | 0.49 | 0.54 | 0.53 | 0.55 | 0.56 |
|  | Scenario2 | **0.66** | 0.50 | 0.62 | 0.65 | 0.62 | 0.63 | 0.60 | 0.54 | 0.57 | **0.62** | 0.61 | 0.56 |
|  | Scenario3 | **0.69** | 0.49 | 0.62 | 0.63 | 0.60 | 0.62 | **0.62** | 0.50 | 0.58 | 0.59 | 0.58 | 0.58 |
|  | Scenario4 | **0.64** | 0.49 | 0.61 | 0.61 | 0.59 | 0.62 | **0.58** | 0.50 | 0.57 | 0.57 | 0.55 | 0.56 |
|  | Scenario5 | 0.59 | 0.52 | 0.59 | 0.59 | 0.59 | **0.60** | 0.54 | 0.50 | 0.54 | **0.57** | **0.57** | 0.55 |
| Small OTU Effect Size* | Scenario1 | **0.63** | 0.50 | 0.57 | 0.55 | 0.53 | 0.56 | 0.55 | 0.50 | 0.53 | 0.52 | 0.53 | **0.56** |
|  | Scenario2 | **0.62** | 0.50 | 0.59 | 0.62 | 0.60 | 0.60 | 0.55 | 0.50 | 0.54 | **0.57** | 0.56 | 0.53 |
|  | Scenario3 | **0.66** | 0.52 | 0.59 | 0.60 | 0.58 | 0.60 | **0.59** | 0.50 | 0.55 | 0.57 | 0.55 | 0.56 |
|  | Scenario4 | **0.60** | 0.49 | 0.59 | 0.59 | 0.57 | 0.59 | 0.55 | 0.50 | 0.54 | **0.56** | 0.53 | 0.54 |
|  | Scenario5 | 0.56 | 0.49 | 0.56 | 0.56 | 0.56 | **0.58** | 0.52 | 0.50 | 0.52 | 0.52 | **0.54** | 0.53 |

* Small OTU effect size is simulated from N(±0.2, 0.04) and N(±0.3,0.06) for continuous and binary outcomes, respectively; large OTU effect size is simulated from N(±0.5, 0.1) and N(±1,0.2) for continuous and binary outcomes, respectively.
