# Supplementary material for: Phylogeny-guided microbiome OTU-specific association test (POST)
Source: Microbiome. 2022 Jun 7;10:86. doi: 10.1186/s40168-022-01266-3 (PMC9171974; doi:10.1186/s40168-022-01266-3)
Supplement: Supplementary file 7 — Additional file 6 Table S2. OTUs significantly associated with preterm birth at FDR level of 0.05. TF: TreeFDR; SO: Single-OTU test implemented by POST with c=0; DE: DESeq2; WR-P: Wilcoxon rank-sum test using proportional data; WR-R: Wilcoxon rank-sum test using CLR transformed data. [file 40168_2022_1266_MOESM6_ESM.docx]

| OTU | FDR Adjusted p-value | | | | | | | | Detected method | Genus/Species | Direction  ** |
| --- | --- | --- | --- | --- | --- | --- | --- | --- | --- | --- | --- |
|  | POST | TF | SO | DE | AB | LD | WR-P* | WR-R* |  |  |  |
| OTU131 | 0.001 | 0.199 | 0.001 | 0.000 | 0.000 | 0.001 | 0.032 | 0.001 | POST/SO/DE/AB/LD /WR-P/WR-R | *Prevotella* sp. | - |
| OTU40 | 0.003 | 0.213 | 0.550 | 0.020 | 0.603 | 0.368 | 0.064 | 0.507 | POST/DE | *Prevotella melaninogenica* | + |
| OTU153 | 0.038 | 0.008 | 0.054 | 0.019 | 0.002 | 0.007 | 0.003 | 0.041 | POST/TF/DE/AB/LD /WR-P/WR-R | *Neisseria* sp. | + |
| OTU72 | 0.361 | 0.008 | 0.146 | 0.094 | 0.002 | 0.029 | 0.000 | 0.133 | TF/AB/LD/WR-P | *Haemophilus parainfluenzae* | + |
| OTU2 | 0.361 | 0.666 | 0.422 | 0.000 | 0.200 | 0.368 | 0.161 | 0.288 | DE | *Lactobacillus crispatus* | - |
| OTU4 | 0.959 | 0.957 | 0.980 | 0.003 | 0.889 | 0.939 | 0.300 | 0.966 | DE | *Veillonellaceae bacterium* | - |
| OTU8 | 0.806 | 0.957 | 0.863 | 0.000 | 0.843 | 0.826 | 0.228 | 0.976 | DE | *Prevotella* sp. | - |
| OTU15 | 0.911 | 0.666 | 0.914 | 0.000 | 0.845 | 0.826 | 0.469 | 0.675 | DE | *Mycoplasma hominis* | + |
| OTU19 | 0.515 | 0.957 | 0.550 | 0.013 | 0.285 | 0.637 | 0.509 | 0.507 | DE | *Staphylococcus aureus* | + |
| OTU71 | 0.904 | 0.920 | 0.951 | 0.004 | 0.903 | 0.932 | 0.813 | 0.675 | DE | *Alloscardovia omnicolens* | + |
| OTU114 | 0.758 | 0.957 | 0.863 | 0.034 | 0.828 | 0.826 | 0.469 | 0.888 | DE | *Lacticaseibacillus rhamnosus* | + |
| OTU126 | 0.399 | 0.434 | 0.447 | 0.019 | 0.360 | 0.200 | 0.004 | 0.420 | DE/WR-P | *Cloacibacterium* sp. | + |
| OTU31 | 0.399 | 0.611 | 0.422 | 0.801 | 0.028 | 0.208 | 0.032 | 0.361 | AB/WR-P | *Staphylococcus anginosus* | + |
| OTU44 | 0.087 | 0.299 | 0.119 | 0.578 | 0.001 | 0.038 | 0.032 | 0.133 | AB/LD/WR-P | *Fusobacterium nucleatum* | + |
| OTU7 | 0.399 | 0.957 | 0.516 | 0.578 | 0.019 | 0.368 | 0.359 | 0.133 | AB | *Clostridiales genomosp. BVAB1* | - |
| OTU11 | 0.758 | 0.957 | 0.741 | 0.633 | 0.617 | 0.368 | 0.034 | 0.661 | WR-P | *Finegoldia magna* | + |
| OTU56 | 0.806 | 0.957 | 0.741 | 0.836 | 0.492 | 0.449 | 0.034 | 0.626 | WR-P | *Anaerococcus hydrogenalis* | + |
| OTU84 | 0.522 | 0.666 | 0.550 | 0.801 | 0.277 | 0.368 | 0.032 | 0.420 | WR-P | *Gemella morbillorum* | + |
| OTU96 | 0.444 | 0.957 | 0.550 | 0.578 | 0.363 | 0.348 | 0.032 | 0.507 | WR-P | *Clostridiales bacterium* | + |

**Table S2. OTUs significantly associated with preterm birth (PTB) at FDR level of 0.05.** TF: TreeFDR; SO: Single-OTU test implemented by POST with $c=0$; DE: DESeq2; AB: ANCOM-BC; LD: LinDA; WR-P: Wilcoxon rank-sum test using proportional data; WR-R: Wilcoxon rank-sum test using CLR transformed data.

*: WR-P and WR-R did not adjust the covariate race.

**: + (and –) indicates that the OTU is positively (and negatively) associated with BV risk from a logistic regression.
