# Supplementary material for: Phylogeny-guided microbiome OTU-specific association test (POST)
Source: Microbiome. 2022 Jun 7;10:86. doi: 10.1186/s40168-022-01266-3 (PMC9171974; doi:10.1186/s40168-022-01266-3)
Supplement: Supplementary file 8 — Additional file 7 Section S1. In this section, we conducted association analysis at the ASV level (i.e., 0% dissimilarity) using the same analysis strategies as described in the main texts for the bacterial vaginosis (BV) and the preterm birth (PTB) association studies. [file 40168_2022_1266_MOESM7_ESM.docx]

**Section S1**

In this section, we conducted association analysis at the ASV level using the same analysis strategies as described in the main texts for the bacterial vaginosis (BV) and the preterm birth (PTB) association studies. That is, we used POST with $c_{max}=0.5$ and the baseline methods, TF, SO, DE, AB, LD, WR-P and WR-R; we included race as a covariate, computed the FDR-adjusted p-values using the TSBH procedure, and selected important ASVs if the FDR-adjusted p-values < 0.05. In this section, we use “ASV” to refer to OTUs formed at 0% dissimilarity, and “OTU” to refer to OTUs formed at 3% dissimilarity.

***ASV-level association study of vaginal microbiome and bacterial vaginosis***

We obtained 4326 ASVs after initial processing using DADA2 and then created the phylogenetic tree using the ASV sequences with *FastTree2*. Because many of the ASVs mapped to the OTUs had low prevalence, we used a less stringent filtering criterion (i.e., abundance < 0.005% and prevalence < 5%) than the OUT-level filters (i.e., abundance < 0.005% and prevalence < 10%) to retain the majority of the ASVs considered in the OUT-level analysis and permit comparisons between ASV-level results and OTU-level results. After filtering, there were 218 ASVs of 39 individuals for BV association analysis.

We summarized the results in an Upset plot (Figure SS1) and listed in Table SS1 the specific information of the ASVs identified by each method. POST, TF, SO, DE, AB, LD, WR-P and WR-R identified 9, 0, 0, 12, 1, 1, 0, and 0 significant ASVs, respectively. We highlighted a few findings: (i) Among the 9 POST-identified ASVs, 3 ASVs were also identified by either DE only or DE+AB+LD. Such patterns are similar to the OTU-level results. (ii) All POST-identified ASVs were mapped to POST-identified OTUs in the OTU-level analysis. There are also POST-identified OTUs that did not have any ASVs identified by POST in the ASV-level analysis, i.e., OTU2; however, we see that ASV20 (mapped to OTU2 and identified by DE here) had an adjusted p-value (0.07) by POST that is close to the FDR threshold. (iii) Among the 9 ASVs that were uniquely identified by DE, ASV15 is mapped to OTU11 which is also identified by DE in the OTU-level analysis. The remaining 8 DE-identified ASVs were mapped to non-significant OTUs in the OUT-level analysis. (iv) Although TF, SO, WR-P and WR-R did not identify any ASVs, SO and WR-R would identify ASV3 (which was found significant by POST, TF, AB and LD) at FDR 0.1 level.


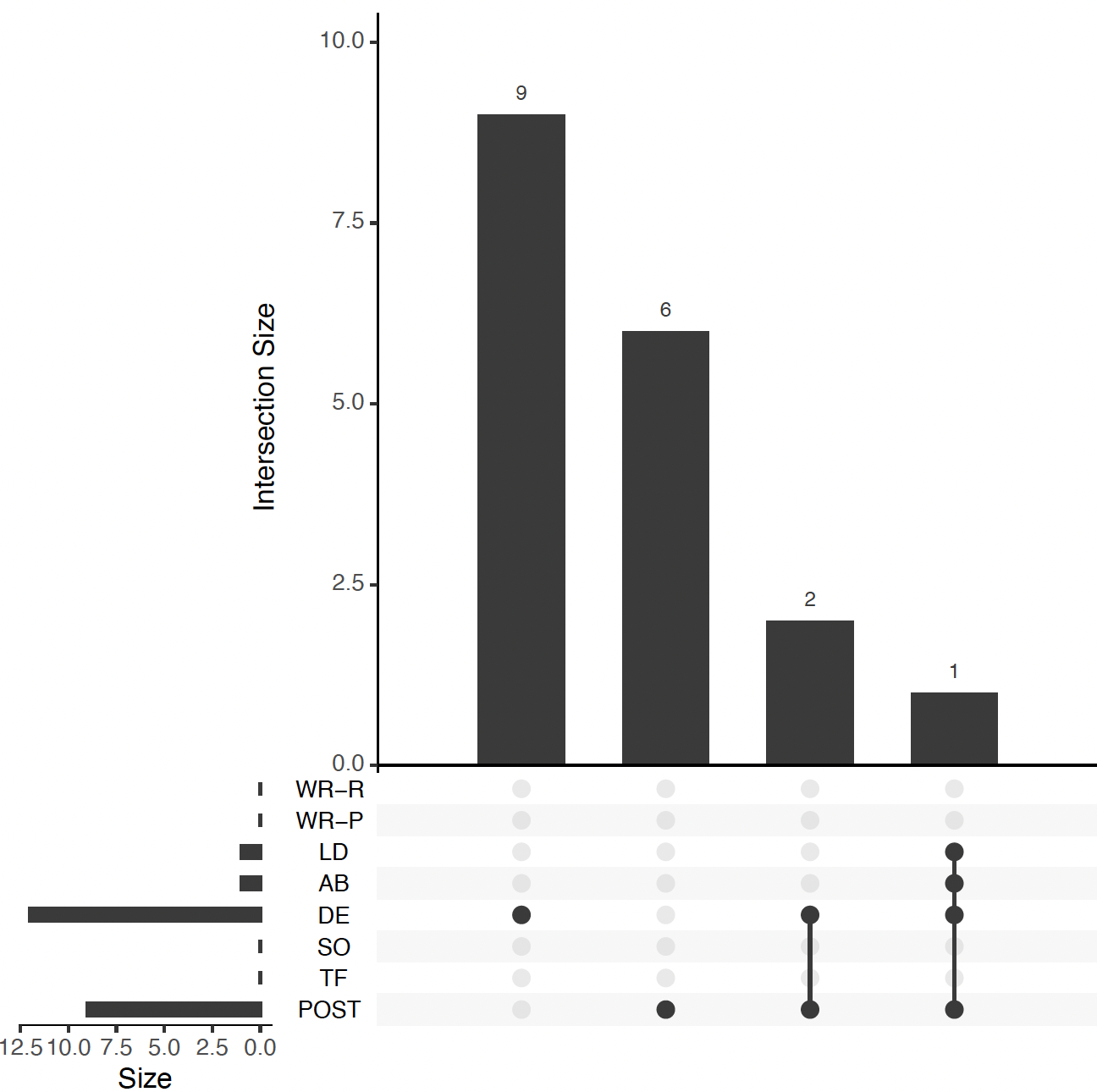


**Figure SS1.** Upset plot of detected ASVs at FDR level of 0.05 for bacterial vaginosis study. TF: TreeFDR; SO: Single-OTU test implemented by POST with c = 0; DE: DESeq2; AB: ANCOM-BC; LD: LinDA; WR-P: Wilcoxon rank-sum test using proportional data; WR-R: Wilcoxon rank-sum test using CLR transformed data.

**Table SS1.** ASVs significantly associated with bacterial vaginosis (BV) at FDR level of 0.05. TF: TreeFDR; SO: Single-OTU test implemented by POST with c = 0; DE: DESeq2; AB: ANCOM-BC; LD: LinDA; WR-P: Wilcoxon rank-sum test using proportional data; WR-R: Wilcoxon rank-sum test using CLR transformed data.

| **ASV** | **FDR Adjusted p-value** | | | | | | | | **Detected method** | **OTU** | **Genus/Species** | **Detected method at OTU level** |
| --- | --- | --- | --- | --- | --- | --- | --- | --- | --- | --- | --- | --- |
|  | **POST** | **SO** | **DE** | **TF** | **AB** | **LD** | **WR-P*** | **WR-R*** |  |  |  |  |
| ASV3 | 0.049 | 0.068 | 0.000 | 0.211 | 0.009 | 0.036 | 0.218 | 0.056 | POST/DE/AB/LD | OTU3 | Lactobacillus crispatus | POST/SO/DE/WR-R/AB/LD |
| ASV5 | 0.049 | 0.527 | 0.000 | 0.488 | 0.372 | 0.512 | 0.284 | 0.116 | POST/DE |  |  |  |
| ASV11 | 0.049 | 0.408 | 0.000 | 0.215 | 0.197 | 0.358 | 0.275 | 0.126 | POST/DE |  |  |  |
| ASV18 | 0.049 | 0.753 | 0.945 | 0.532 | 0.555 | 0.620 | 1.000 | 0.262 | POST |  |  |  |
| ASV88 | 0.049 | 0.975 | 0.493 | 0.329 | 0.978 | 0.961 | 0.483 | 0.919 | POST | OTU58 | Lactobacillus sp. | POST |
| ASV100 | 0.049 | 0.524 | 0.770 | 0.561 | 0.335 | 0.446 | 0.515 | 0.215 | POST | OTU66 | Lactobacillus sp. | POST |
| ASV138 | 0.049 | 0.990 | 0.843 | 0.211 | 0.994 | 0.961 | 0.400 | 0.919 | POST | OTU90 | Lactobacillus sp. | POST |
| ASV124 | 0.049 | 0.949 | 0.891 | 0.930 | 0.906 | 0.913 | 0.449 | 0.678 | POST | OTU82 | Lactobacillus gasseri | POST |
| ASV8 | 0.049 | 0.753 | 0.099 | 0.779 | 0.662 | 0.569 | 0.727 | 0.449 | POST | OTU7 | Lactobacillus jensenii | POST |
| ASV20 | 0.070 | 0.789 | 0.000 | 0.530 | 0.662 | 0.794 | 0.636 | 0.252 | DE | OTU2 | Lactobacillus iners | POST |
| ASV87 | 0.970 | 0.975 | 0.000 | 0.272 | 0.978 | 0.978 | 0.288 | 0.744 | DE | OTU33 | Clostridium celatum | N/A |
| ASV169 | 0.975 | 0.990 | 0.000 | 0.354 | 0.994 | 0.969 | 0.286 | 0.763 | DE | OTU9 | Gardnerella vaginalis | N/A |
| ASV61 | 0.914 | 0.975 | 0.000 | 0.376 | 0.978 | 0.961 | 0.480 | 0.561 | DE | OTU5 | Fannyhessea vaginae | N/A |
| ASV77 | 0.835 | 0.975 | 0.000 | 0.186 | 0.957 | 0.961 | 0.256 | 0.561 | DE | OTU4 | Veillonellaceae sp. | N/A |
| ASV80 | 0.727 | 0.919 | 0.028 | 0.160 | 0.765 | 0.907 | 0.218 | 0.603 | DE | OTU53 | Dialister micraerophilus | N/A |
| ASV51 | 0.975 | 0.975 | 0.009 | 0.607 | 0.978 | 0.961 | 0.320 | 0.848 | DE | OTU31 | Prevotella timonensis | N/A |
| ASV44 | 0.786 | 0.975 | 0.000 | 0.280 | 0.953 | 0.961 | 0.256 | 0.621 | DE | OTU8 | Prevotella sp. | N/A |
| ASV15 | 0.806 | 0.919 | 0.000 | 0.316 | 0.848 | 0.907 | 0.225 | 0.726 | DE | OTU11 | Prevotella sp. | DE |

* WR-P and WR-R did not adjust for race.

***ASV-level association study of vaginal microbiome and preterm birth***

We obtained 1537 ASVs after initial processing using DADA2 and then created the phylogenetic tree using the ASV sequences with *FastTree2*. Because many of the ASVs mapped to the OTUs had low abundance, we used a less stringent filtering criterion (i.e., abundance < 0.003% and prevalence < 10%) than the OUT-level filters (i.e., abundance < 0.005% and prevalence < 10%) to retain the majority of the ASVs considered in the OUT-level analysis and facilitate the comparisons between ASV-level results and OTU-level results. After filtering, there were 161 ASVs of 39 individuals for PTB association.

We summarized the results in an Upset plot (Figure SS2) and listed in Table SS2 the specific information of the ASVs identified by each method. POST, TF, SO, DE, AB, LD, WR-P and WR-R, AB and LD identified 3, 3, 3, 18, 10, 5, 7 and 1 significant ASVs, respectively. (i) Two of the 3 POST-identified ASVs (i.e., ASV220 and ASV376) were also identified by several other methods. ASV57, identified by POST only, was mapped to OTU40, which is identified by POST and DE in the OTU-level analysis. (ii) POST did not identify 4 ASVs that were found significant by more than one baseline methods (i.e., ASV279, ASV273, ASV107 and ASV164). Such patterns are similar to the OTU-level results, i.e., POST performed similarly to the non-tree, CLR based baseline methods, all of which had fewer significant findings. (iii) Similar to the OTU-level analysis, DE uniquely identified many ASVs; 6 of these 15 ASVs were mapped to OTUs that were uniquely identified by DE at the OUT-level analysis.


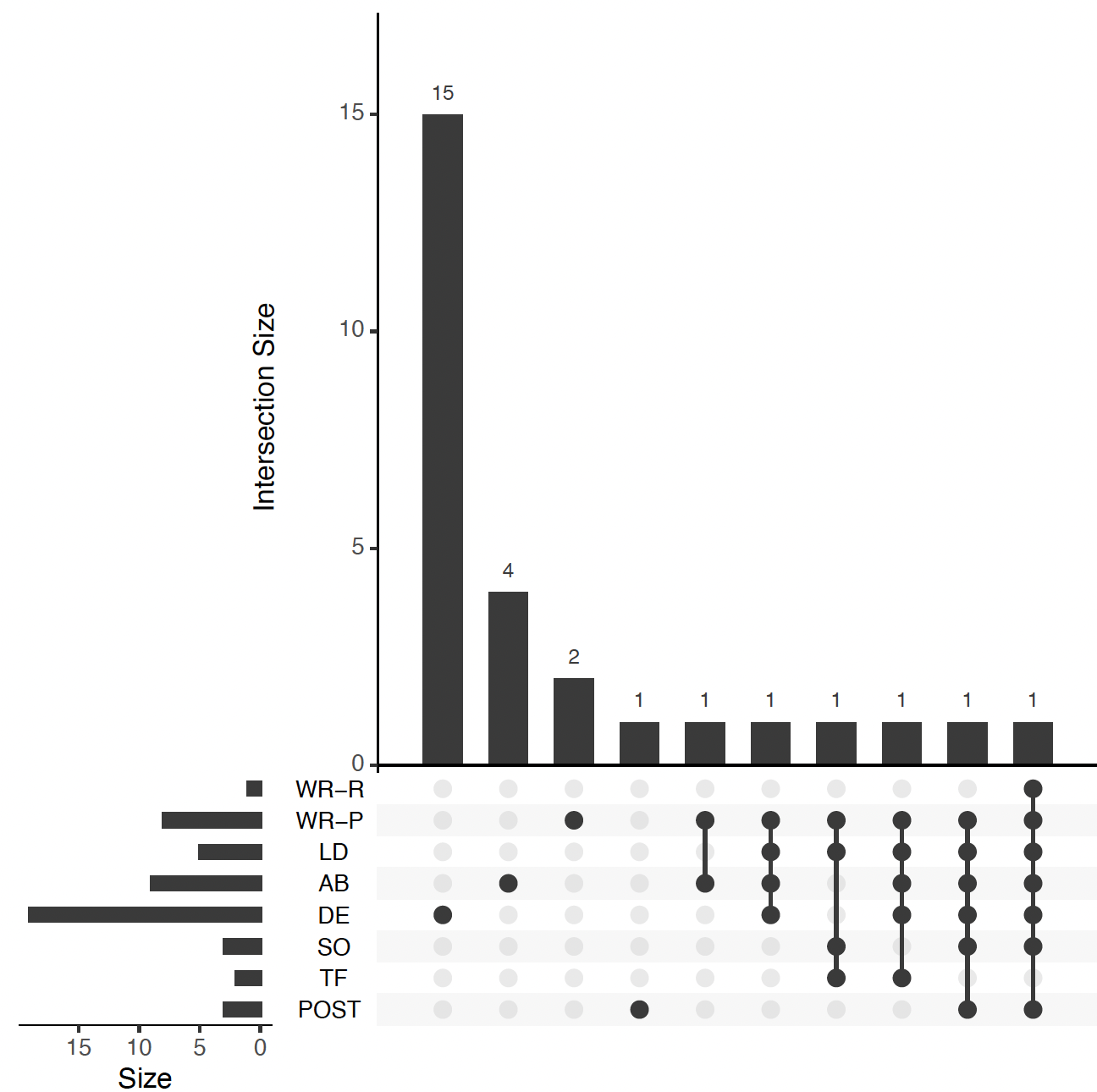


**Figure SS2.** Upset plot of detected ASVs at FDR level of 0.05 for preterm birth study. TF: TreeFDR; SO: Single-OTU test implemented by POST with c = 0; DE: DESeq2; AB: ANCOM-BC; LD: LinDA; WR-P: Wilcoxon rank-sum test using proportional data; WR-R: Wilcoxon rank-sum test using CLR transformed data.

**Table SS2**. ASVs significantly associated with preterm birth at FDR level of 0.05. TF: TreeFDR; SO: Single-OTU test implemented by POST with c = 0; DE: DESeq2; AB: ANCOM-BC; LD: LinDA; WR-P: Wilcoxon rank-sum test using proportional data; WR-R: Wilcoxon rank-sum test using CLR transformed data.

| **ASV** | **FDR Adjusted p-value** | | | | | | | | **Detected method** | **OTU** | **Genus/Species** | **Detected method at OTU level** |
| --- | --- | --- | --- | --- | --- | --- | --- | --- | --- | --- | --- | --- |
|  | **POST** | **SO** | **DE** | **TF** | **AB** | **LD** | **WR-P*** | **WR-R*** |  |  |  |  |
| ASV220 | 0.000 | 0.000 | 0.000 | 0.225 | 0.000 | 0.000 | 0.026 | 0.002 | POST/SO/DE/AB/LD/WR-P/WR-R | OTU131 | Prevotella sp. | POST/SO/DE/AB/LD/WR-P/WR-R |
| ASV57 | 0.001 | 0.598 | 0.085 | 0.209 | 0.312 | 0.410 | 0.056 | 0.468 | POST | OTU40 | Prevotella melaninogenica | POST/DE |
| ASV376 | 0.021 | 0.021 | 0.003 | 0.091 | 0.007 | 0.005 | 0.002 | 0.097 | POST/SO/DE/AB/LD/WR-P | OTU197 | Pseudomonas sp. | - |
| ASV279 | 0.061 | 0.021 | 0.059 | 0.005 | 0.059 | 0.025 | 0.001 | 0.298 | SO/TF/LD/WR-P | OTU74 | Veillonella sp. | - |
| ASV262 | 0.093 | 0.541 | 0.518 | 0.212 | 0.259 | 0.410 | 0.044 | 0.475 | WR-P |  |  |  |
| ASV273 | 0.093 | 0.153 | 0.001 | 0.068 | 0.007 | 0.025 | 0.017 | 0.097 | DE/AB/LD/WR-P | OTU153 | Neisseria sp. | POST/TF/DE/ AB/LD/WR-P/WR-R |
| ASV2 | 0.111 | 0.463 | 0.001 | 0.678 | 0.312 | 0.446 | 0.254 | 0.298 | DE | OTU2 | Lactobacillus crispatus | DE |
| ASV11 | 0.111 | 0.533 | 0.001 | 0.635 | 0.058 | 0.410 | 0.367 | 0.229 | DE |  |  |  |
| ASV107 | 0.185 | 0.154 | 0.032 | 0.005 | 0.000 | 0.025 | 0.001 | 0.135 | DE/AB/LD /TF/WR-P | OTU72 | Haemophilus parainfluenzae | TF/AB/LD/WR-P |
| ASV97 | 0.412 | 0.857 | 0.038 | 0.982 | 0.887 | 0.745 | 0.880 | 0.468 | DE | OTU67 | Lactobacillus coleohominis | - |
| ASV27 | 0.364 | 0.463 | 0.834 | 0.982 | 0.007 | 0.377 | 0.074 | 0.334 | AB | OTU12 | Prevotella sp. | - |
| ASV125 | 0.436 | 0.945 | 0.002 | 0.797 | 0.734 | 0.927 | 0.206 | 0.993 | DE |  |  |  |
| ASV24 | 0.572 | 0.621 | 0.022 | 0.982 | 0.764 | 0.737 | 0.518 | 0.475 | DE | OTU19 | Staphylococcus aureus | DE |
| ASV203 | 0.607 | 0.758 | 0.013 | 0.982 | 0.798 | 0.646 | 0.776 | 0.322 | DE |  |  |  |
| ASV10 | 0.980 | 0.965 | 0.000 | 0.982 | 0.843 | 0.927 | 0.301 | 0.994 | DE | OTU8 | Prevotella sp. | - |
| ASV237 | 0.980 | 0.965 | 0.002 | 0.982 | 0.847 | 0.927 | 0.637 | 0.821 | DE | OTU39 | Bifidobacterium dentium | - |
| ASV106 | 0.980 | 0.967 | 0.001 | 0.491 | 0.887 | 0.934 | 0.858 | 0.707 | DE | OTU71 | Alloscardovia omnicolens | DE |
| ASV277 | 0.980 | 0.965 | 0.001 | 0.539 | 0.801 | 0.927 | 0.275 | 0.924 | DE | OTU56 | Anaerococcus hydrogenalis | WR-P |
| ASV171 | 0.980 | 0.758 | 0.005 | 0.982 | 0.798 | 0.646 | 0.760 | 0.298 | DE |  |  |  |
| ASV195 | 0.980 | 0.965 | 0.014 | 0.678 | 0.887 | 0.927 | 0.747 | 0.665 | DE |  |  |  |
| ASV20 | 0.980 | 0.967 | 0.000 | 0.654 | 0.887 | 0.927 | 0.563 | 0.665 | DE | OTU15 | Mycoplasma hominis | - |
| ASV5 | 0.980 | 0.972 | 0.005 | 0.982 | 0.887 | 0.955 | 0.394 | 0.993 | DE | OTU4 | Veillonellaceae bacterium | DE |
| ASV39 | 0.980 | 0.967 | 0.022 | 0.773 | 0.887 | 0.955 | 0.569 | 0.924 | DE | OTU27 | Corynebacterium sp. | - |
| ASV164 | 0.151 | 0.250 | 0.698 | 0.427 | 0.003 | 0.088 | 0.024 | 0.298 | AB/WR-P | OTU107 | Streptococcus thermophilus | - |
| ASV45 | 0.171 | 0.463 | 0.698 | 0.209 | 0.007 | 0.301 | 0.052 | 0.298 | AB | OTU31 | Staphylococcus anginosus | AB/WR-P |
| ASV60 | 0.750 | 0.576 | 0.627 | 0.635 | 0.008 | 0.410 | 0.152 | 0.591 | AB | OTU42 | Prevotella buccalis | - |
| ASV42 | 0.980 | 0.621 | 0.854 | 0.797 | 0.026 | 0.420 | 0.104 | 0.542 | AB | OTU22 | Anaerococcus sp. | - |
| ASV93 | 0.093 | 0.648 | 0.817 | 0.403 | 0.281 | 0.558 | 0.024 | 0.468 | WR-P | OTU64 | Veillonella sp. | - |

* WR-P and WR-R did not adjust for race.
