# Supplementary material for: Phylogeny-guided microbiome OTU-specific association test (POST)
Source: Microbiome. 2022 Jun 7;10:86. doi: 10.1186/s40168-022-01266-3 (PMC9171974; doi:10.1186/s40168-022-01266-3)
Supplement: Supplementary file 9 — Additional file 8 Table S3. The AUC using different pseudo-counts in Simulation A. The AUCs based on pseudo-count 0.5 are very close to the AUCs based on pseudo-count 1 across different scenarios and effect sizes. [file 40168_2022_1266_MOESM8_ESM.docx]

**Table S3.** The AUC using different pseudo-counts in Simulation A. The AUCs based on pseudo-count 0.5 are very close to the AUCs based on pseudo-count 1 across different scenarios and effect sizes.

| **Effect size** | **Scenario** | **Pseudo-count 0.5** | **Pseudo-count 1** |
| --- | --- | --- | --- |
| Large effect size* | 1 | 0.80 | 0.82 |
|  | 2 | 0.88 | 0.89 |
|  | 3 | 0.88 | 0.89 |
|  | 4 | 0.78 | 0.79 |
|  | 5 | 0.66 | 0.67 |
| Small effect size* | 1 | 0.72 | 0.74 |
|  | 2 | 0.76 | 0.78 |
|  | 3 | 0.76 | 0.78 |
|  | 4 | 0.69 | 0.70 |
|  | 5 | 0.59 | 0.59 |

*Small effect size is from N(±1,1) and large effect size is from N(±2,1).
